# A comparative analysis of transcriptomics of newly diagnosed multiple myeloma: exploring drug repurposing

**DOI:** 10.3389/fonc.2024.1390105

**Published:** 2024-04-16

**Authors:** Angelos Giannakoulas, Marios Nikolaidis, Grigorios D. Amoutzias, Nikolaos Giannakoulas

**Affiliations:** ^1^ Department of Hematology, Faculty of Medicine, School of Health Sciences, University of Thessaly, Larissa, Greece; ^2^ Bioinformatics Laboratory, Department of Biochemistry & Biotechnology, School of Health Sciences, University of Thessaly, Larissa, Greece

**Keywords:** multiple myeloma, NDMM, gene expression profiling, drug repurposing, transcriptomics, ADM, adrenomedullin, PURPL

## Abstract

Multiple myeloma (MM) is an incurable malignant plasma cell disorder characterized by the infiltration of clonal plasma cells in the bone marrow compartment. Gene Expression Profiling (GEP) has emerged as a powerful investigation tool in modern myeloma research enabling the dissection of the molecular background of MM and allowing the identification of gene products that could potentially serve as targets for therapeutic intervention. In this study we investigated shared transcriptomic abnormalities across newly diagnosed multiple myeloma (NDMM) patient cohorts. In total, publicly available transcriptomic data of 7 studies from CD138+ cells from 281 NDMM patients and 44 healthy individuals were integrated and analyzed. Overall, we identified 28 genes that were consistently differentially expressed (DE) between NDMM patients and healthy donors (HD) across various studies. Of those, 9 genes were over/under-expressed in more than 75% of NDMM patients. In addition, we identified 4 genes (MT1F, PURPL, LINC01239 and LINC01480) that were not previously considered to participate in MM pathogenesis. Meanwhile, by mining three drug databases (ChEMBL, IUPHAR/BPS and DrugBank) we identified 31 FDA-approved and 144 experimental drugs that target 8 of these 28 over/under-expressed MM genes. Taken together, our study offers new insights in MM pathogenesis and importantly, it reveals potential new treatment options that need to be further investigated in future studies.

## 1 Introduction

Multiple myeloma (MM) is a malignant plasma cell disorder characterized by the infiltration of clonal plasma cells (PCs) in the bone marrow. It is the second most frequent hematologic malignancy with an incidence of 5/100.000 in Europe (1). It mainly affects the elderly with average age of diagnosis at 70 years old and is slightly more common in male than female (2). It arises from a premalignant condition termed monoclonal gammopathy of undetermined significance (MGUS), which is observed in approximately 3% of the population over 50 years old (3). The progression risk from MGUS to symptomatic MM or other lymphoproliferative disorders accounts for 1% per year (4). Hallmark features of symptomatic MM include anemia, hypercalcemia, renal insufficiency, osteolytic bone lesions and increased vulnerability to infections (5).

Therapeutic advances over the past decades have significantly extended survival and improved quality of life of MM patients. At the moment, more than 20 drugs are available for the management of multiple myeloma (6). The treatment decision is currently based on age, performance status and risk stratification (7). Age-standardized five-year survival has increased from 41% to 69% for patients under 69 years old and from 23% to 47% for patients between 70 and 79 years old (8). Despite significant progress, MM remains incurable and inevitably all patients will experience relapses and eventually develop refractory disease. Further research is needed to improve our understanding of MM complex biology, which will enable the identification of novel therapeutic targets and the subsequent development of more effective treatments.

Intensive research of the past decades has revealed that MM is a highly heterogenous disease with several molecular subtypes (9). According to the International Myeloma Working Group, the major subgroups are the hyperdiploid, the t(4;14)(p16;q32), the t(11;14)(q13;q32), the t(14;16)(q32;q23) and the unclassified type (10). Each subgroup is characterized by distinct genetic and molecular alterations resulting in unique clinicopathological features, prognostic implications and treatment outcomes. This diversity across MM subgroups is the main reason for failure of treatments that selectively interfere with specific MM targets. Nevertheless, exploration of shared abnormal patterns within or even across myeloma subgroups can surpass MM variability and identify unifying molecular abnormalities or abnormalities shared by members of a certain subgroup, which could guide precision-based drug administration, drug development or even drug repurposing.

Gene expression profiling (GEP) has emerged as a powerful investigation tool in modern myeloma research. Many GEP studies have been conducted on MM with the purpose to reveal distinct subgroups and to identify gene signatures that predict poor treatment outcomes and inferior overall survival (11, 12). However, so far, no thorough comparative analysis of the publicly available datasets has been conducted to identify genes that are consistently over/under-expressed across newly diagnosed multiple myeloma (NDMM) patient cohorts.

Herein, using publicly available transcriptomic data from CD138+ cells from 2 bulk RNA-seq and 5 Affymetrix Chip studies of NDMM patients against their controls we investigated gene expression profiles aiming to reveal transcriptomic similarities across NDMM patients which could potentially serve as therapeutic targets (13–19). We identified 28 genes that were consistently differentially expressed (DE) across various studies, with several of them not being considered as ‘key’ genes in MM previously. Furthermore, we mined drug databases to identify already approved drugs for other diseases that target these genes and thus could be re-purposed for MM.

## 2 Materials and methods

### 2.1 Data mining

The Gene Expression Omnibus (GEO) database was mined for publicly available transcriptomic data of CD138+ cells from NDMM patients and healthy donors (HD) (20). The search strategy contained the MESH TERMS ‘‘Multiple Myeloma’’ AND ‘‘Expression profiling by high throughput sequencing’’ OR ‘‘Expression profiling by array’’. Datasets were considered eligible for this study if they fulfilled the following predefined inclusion criterion: each candidate dataset ought to contain bulk RNA-seq or microarray-based transcriptomic data from purified PCs derived from bone marrow aspirates, both from HD and from NDMM patients. An initial search identified 779 datasets. Each dataset was screened for relevance and eligibility. Irrelevant datasets, duplicate datasets, non-coding RNA-based datasets, datasets with unavailable raw data and datasets where patients could not be clearly distinguished from healthy individuals, based on UMAP clustering (at the GEO website), were excluded. Overall, 2 bulk RNA-seq datasets (accession numbers GSE153380 and GSE175384) and 5 microarray-based datasets (accession numbers GSE116294, GSE6691, GSE47552, GSE6477 and GSE16558) were included in our analyses (**Supplementary Figure 1**).

### 2.2 Samples and patient characteristics

Prior to gene expression analysis we evaluated the credibility of each NDMM and HD sample of the 7 datasets by using UMAP plots at the GEO website, in order to assess whether samples from NDMM patients were clustering together and not clustering with samples from HD and vice versa. Thus, we excluded 3 samples derived from HD of the GSE6477 dataset and 1 sample derived from a NDMM patient of the GSE16558 dataset. In total, transcriptomic data from 281 NDMM patients and 44 healthy individuals were obtained and re-analyzed (**Supplementary Table 1**). Cytogenetic abnormalities of each patient cohort are summarized in **Supplementary Table 2**.

### 2.3 Data processing

#### 2.3.1 Bulk RNA seq datasets

We downloaded the raw SRA data from the GEO studies GSE153380 and GSE175384. The raw data from both studies were analyzed with FastQC and trimmed with Trimmomatic (21, 22). The trimmomatic parameters were set to ILLUMINACLIP : TruSeq3-PE-2:2:30:10 SLIDINGWINDOW:4:30 MINLEN:70 for GSE153380 and ILLUMINACLIP : TruSeq3-SE:2:30:10 SLIDINGWINDOW:4:30 MINLEN:50 for GSE175384. Then, the trimmed reads were aligned to the reference human genome (version GRCh38.110) from ENSEMBL with STAR and the counts for each gene were obtained (23). For each study the gene expression counts were cross-sample normalized using the TMM method to enable the comparison of NDMM patients and HD. Differential gene expression analysis was conducted with DESeq2 and EdgeR on the raw gene expression counts (24, 25). For a gene to be considered as DE, it should have a P-value (after multiple-testing correction) less than 0.05 and absolute log2 fold-change more than or equal to 2.

#### 2.3.2 Microarray-based datasets

Each microarray-based dataset was analyzed through NCBI GEO2R, comparing the NDMM and HD samples with default parameters (26). DE genes were considered those with P-value (after multiple-testing correction) less than 0.05 and absolute log2 fold-change more than or equal to 2. Gene symbols from the microarrays were mapped to the reference ENSEMBL symbols through HUGO (27). The DE genes from the microarray datasets were subsequently compared with the lists of DE genes from the RNA-seq datasets (identified by DESeq2).

### 2.4 Statistical analysis

All statistical analyses were conducted with the GraphPad Prism 10 software. Results were considered significant when P-values were less than 0.05. Trimmed mean of M (TMM) values were used as expression measures to compare differences in gene expression between subgroups (28). Normality of distributions was assessed with the Shapiro-Wilk test. For non-parametric metrical data, the Mann Whitney U test was used to compare differences between 2 groups and the Kruskal-Wallis test was used to compare differences between 3 or more groups. One sample T-Test and Wilcoxon signed-rank test were used to compare a known mean or a known median of the HD population against the value of a NDMM patient, when appropriate. Graphs were illustrated with GraphPad Prism 10 and R software. Values were logarithmically transformed prior plotting to ease visualization.

## 3 Results

### 3.1 Consistently over/under-expressed genes across several studies

First, we identified in each of the seven transcriptomic studies those genes whose expression was significantly differentiated between NDMM patients and HD. We applied a stringent threshold of absolute fold change ≥ 4 (log2fc ≥ 2) and P-adj < 0.05 in order to obtain those genes with significant change. Next, we integrated the lists of DE genes from each individual dataset. We identified 91 genes that were observed to be differentially expressed between NDMM patients and healthy donors in at least 3 of the 7 datasets (**Supplementary Table 3**). By applying this very strict criterion of 3 experiments, we ensured that any false positives due to batch effects or other sources of variability between samples or subgroups in any of the individual studies would be filtered out. To further filter out our results, we excluded genes with no functional significance. Thus, 31 of the above 91 genes coding for immunoglobulin variants or HLA antigens were excluded. Subsequently, the 60 remaining genes were all manually inspected and 32 of them with no current bibliographic evidence of involvement in MM or any other malignant disorder were also excluded. Nevertheless, these 32 genes are not necessarily irrelevant to MM and may comprise targets for future studies. After all these filtering criteria, 28 MM-associated genes were identified with 22 of them being over-expressed in the NDMM group (**Table 1**). For 2 genes (CXCL12 and VCAM1) there was a discrepancy of fold change values between studies. Both genes were upregulated in the NDMM group of 1 RNA-seq study but downregulated in the NDMM group of 4 Affymetrix gene chip studies. We consulted literature and designated these 2 genes as upregulated, which is in accordance with current bibliographic evidence (29–32). Reassuringly, 13/28 genes are already known to play an important role in MM biology. These genes are HGF, DKK1, CCND1, PTPRC, CD19, CCL3, CD81, EDNRB, CD27, VCAM1, CXCL12, LAMP5 and ST3GAL6. Interestingly, the relevance of the other 15 genes to MM is less well documented and thus they may serve as new disease markers or even therapeutic targets. These 15 genes are TSPAN7, DUSP4, ADM, GADD45A, PRDM5, IFI6, NDNF, PRR15, BTBD3, TGFBI, IFITM1, PURPL, MT1F, LINC01239 and LINC01480.

**Table 1:** List of the 28 overlapping differentially expressed genes across datasets.

Gene Name	Gencodev44_ensembl	Mean log2fc value across datasets	Number of Individual datasets that identified each gene as differentially expressed
ADM	ENSG00000148926	3.1	4
BTBD3	ENSG00000132640	3.2	3
CCL3	ENSG00000277632	2.7	3
CCND1	ENSG00000110092	3.4	3
CD19	ENSG00000177455	-2.8	4
CD27	ENSG00000139193	-2.9	7
CD81	ENSG00000110651	-2.3	4
CXCL12	ENSG00000107562	3.4	5
DKK1	ENSG00000107984	4.6	5
DUSP4	ENSG00000120875	2.7	3
EDNRB	ENSG00000136160	5.3	4
GADD45A	ENSG00000116717	2.5	5
HGF	ENSG00000019991	3.7	6
IFI6	ENSG00000126709	2.3	4
IFITM1	ENSG00000185885	2.2	3
LAMP5	ENSG00000125869	4.2	3
LINC01239	ENSG00000234840	4.5	3
LINC01480	ENSG00000270164	2.3	3
MT1F	ENSG00000198417	-3.1	3
NDNF	ENSG00000173376	2.9	4
PRDM5	ENSG00000138738	2.5	3
PRR15	ENSG00000176532	3.1	3
PTPRC	ENSG00000081237	-2.6	4
PURPL	ENSG00000250337	4.4	3
ST3GAL6	ENSG00000064225	2.7	3
TGFBI	ENSG00000120708	-3	3
TSPAN7	ENSG00000156298	4.2	3
VCAM1	ENSG00000162692	3.8	5

**Table 1** summarizes the list of 28 genes that were consistently differentially expressed between newly diagnosed multiple myeloma patients and healthy donors across various studies. It indicates the mean log2fc value of each gene across datasets and the number of individual datasets that identified each gene as differentially expressed (absolute log2fc ≥2 and P-adj <0.05). Genes are presented with alphabetical order. Positive log2fc indicates upregulation in the NDMM group.

### 3.2 Functional characterization of the 28 MM genes

**Figure 1:**
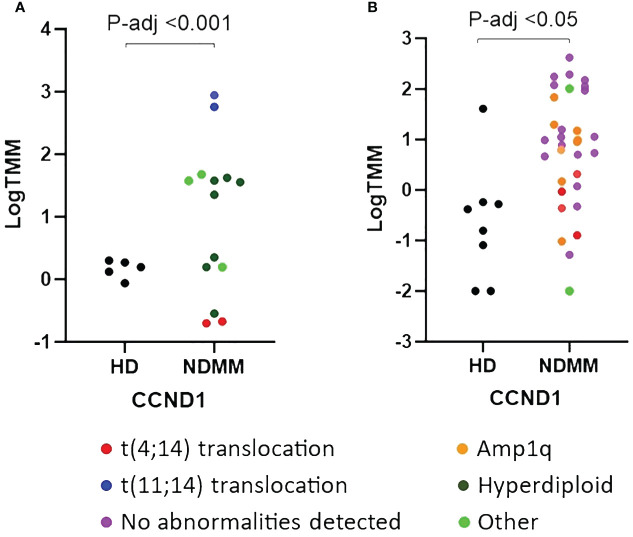
Comparison of relative expression of CCND1 gene between newly diagnosed multiple myeloma (NDMM) patients and healthy donors (HD) from GSE153380 **(A)** and GSE175384 **(B)** datasets. P-adj values were calculated with DESeq2. Cytogenetic abnormalities are included using a color code.

**Figure 2:**
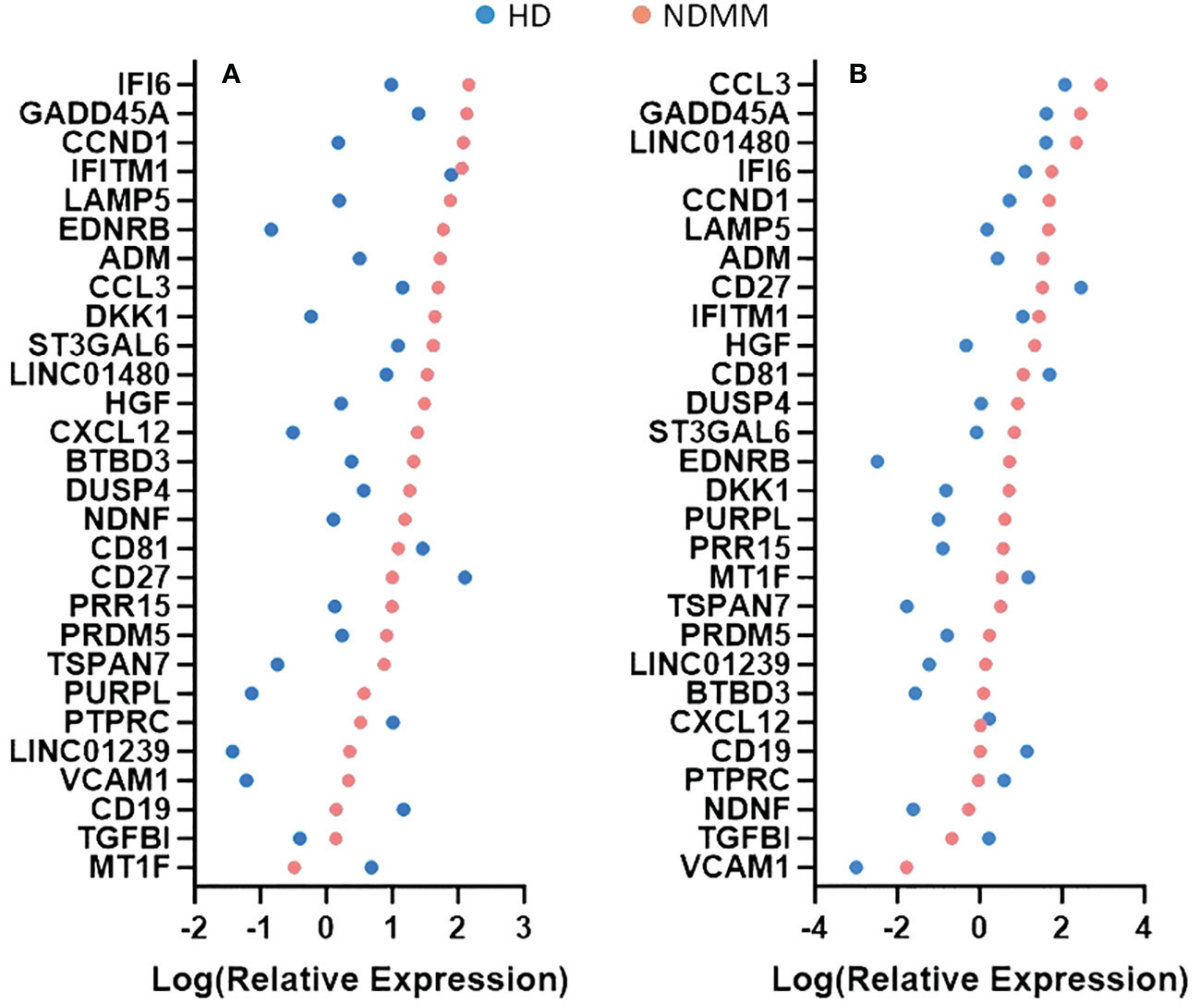
Comparison of relative expression measures of the 28 genes between healthy donors (HD) and newly diagnosed multiple myeloma (NDMM) patients. TMM values were obtained from GSE153380 **(A)** and GSE175384 **(B)** datasets. The mean TMM value of each gene of each group was calculated, log-transformed and plotted. A color code is used to separate the HD group from the NDMM group. When a gene’s mean TMM value equaled to 0, we plotted that value as 0.001. Genes are presented according to the intensity of their expression measures.

We further investigated the role of the 28 genes that were consistently differentially expressed across various studies. Based on their function, 9 of them modulate cell cycle (CCND1, HGF, LAMP5, EDNRB, DUSP4, IFI6, MT1F, PURPL and TGFBI) (33–41), 7 of them are involved in cell-cell interactions and signaling (ST3GAL6, TSPAN7, VCAM1, PTPRC, CD19, CD27 and CD81) (42–48), 3 of them contribute to myeloma bone disease (MBD) (DKK1, CCL3 and CXCL12) (49–51), 2 of them promote angiogenesis (ADM and NDNF) (52, 53) and 7 of them are of unknown functional role (PRDM5, BTBD3, PRR15, LINC01480, IFITM1, LINC01239 and GADD45A). When considering the mean P-adj value across studies, the DKK1 gene, coding for a soluble inhibitor of WNT signaling, and the ADM gene, coding for a vascularization peptide that enhances MM-driven neo-angiogenesis, were among the most significant genes (**Supplementary Table 4**) (49, 52). When considering the mean log2fc value, the EDNRB gene, coding for a G-protein coupled receptor, was the most over-expressed gene while the MT1F gene, coding for metallothionein 1F, was the most under-expressed gene (39, 54). When considering the number of individual datasets that identified a gene as significantly over/under-expressed, the HGF gene was found significantly upregulated in 6 studies whereas the CD27 gene was found significantly downregulated in all of the 7 studies. As expected, CCND1 spiked expression was detected in patients harboring the t(11;14) translocation (55). Interestingly, CCND1 gene was additionally over-expressed by NDMM patients without chromosome 11 abnormalities indicating that cyclin D1 involvement in MM pathogenesis exceeds t(11;14) translocation and trisomy 11 (**Figure 1**). Other oncogenes were DUSP4 and LAMP5 (35, 56–58). Both of them were over-expressed in the NDMM group of the RNA-seq studies and one Affymetrix study. Other genes of particular interest were ST3GAL6 and TSPAN7, both mediating MM homing and migration (42, 43). Even though the fold change and the p values of all the 28 genes were significant, their expression measures were highly variable (**Supplementary Figure 2**). Of note, IFI6, GADD45A, CCL3 and LINC01480 were among the genes with the highest TMM values while TGFBI, VCAM1 and CD19 were among the genes with the lowest values (**Figure 2**). Next, we calculated the number of NDMM patients that significantly over/under-expressed each of the 28 genes. We compared the expression value of each gene of each patient with the expression values of the HD in each of the 2 RNA-seq studies, separately. A total of 9 genes (DKK1, PTPRC, CD19, CD27, MT1F, EDNRB, PURPL, LINC01239 and LINC01480) were over/under-expressed in more than 75% of NDMM patients of both RNA-seq studies (**Supplementary Table 5**). Subgroup analysis of the 2 RNA-seq studies did not reveal any gene whose up/down-regulation was restricted in a specific subgroup. However, we did observe elevated expression of LINC01480 gene in patients harboring t(4;14) translocation, but more studies with higher number of patients are needed to assess the significance of this finding (**Supplementary Figure 3**).

### 3.3 Novel multiple myeloma genes

Our comparative analysis identified 11 genes whose relevance to MM is less well characterized and 4 genes (MT1F, PURPL, LINC01239 and LINC01480) that were not previously considered to participate in MM pathogenesis. The putative tumor suppressor gene MT1F was downregulated in the NDMM group of 3 datasets with a mean log2fc value of 3.1 (59, 60). In contrast, 3 genes encoding long non-coding RNA molecules (PURPL, LINC01239 and LINC01480) were upregulated in the NDMM group of the 2 RNA-seq studies and 1 Affymetrix study (40, 61, 62). It is worth mentioning that only 3 of the 7 datasets (GSE152280, GSE175384 and GSE116294) had the capacity to identify expression of non-coding RNA genes due to GEP platform usage and design of those studies. Interestingly, the 3 long non-coding RNA molecules were expressed by a large percentage of patients (88%, 85%, 83% respectively) whereas the expression of PURPL and LINC01239 was almost absent in healthy donors. Mechanistically, of the 3 non-coding RNA genes, PURPL is the only one that has been thoroughly studied and is implicated in the pathogenesis of several solid malignancies by interfering with crucial signaling cascades and pathways (40, 63–66).

### 3.4 Genes mediating malignant transformation of PCs

Since multiple myeloma is preceded by MGUS and smoldering myeloma (SMM), we hypothesized that genes mediating malignant transformation of normal PCs should be over/under-expressed in MM precursor stages. We compared the 28 MM genes with DE genes between normal PCs and PCs from patients with MGUS and SMM (**Supplementary Data**). Cross-comparison revealed an overlap of 10 genes implying that these genes might contribute to MM development. More precisely, 7 (HGF, CCND1, GADD45A, DUSP4, NDNF, BTBD3 and ST3GAL6) and 3 (CD81, CD27 and PTPRC) of the 28 genes were found upregulated and downregulated respectively in patients with premalignant plasma cell disorders compared to healthy individuals.

### 3.5 Druggable gene products

Prompted by the necessity for additional treatment options for MM patients, we investigated whether existing FDA-approved or experimental drugs target any of the 22 over-expressed MM gene products that our study identified. Towards this end, we mined Drugbank, ChEMBL and IUPHAR/PBS databases (67–69). Additionally, for genes encoding soluble molecules we also evaluated the existence of drugs inhibiting their binding receptors. Our search revealed that 31 FDA-approved and 144 experimental drugs target 8 of the 22 MM products (HGF, CCND1, DKK1, EDNRB, VCAM1, ADM, CCL3, CXCL12) (**Supplementary Table 6**). Afterwards, we focused on the 31 FDA-approved drugs and their targets for further investigation. By inspecting the current literature, we examined which of them target an MM gene product as a primary mechanism of action (MOA) and found that 14/31 drugs antagonize an MM gene product as a dominant MOA. Interestingly, of the 14 short-listed drugs only 2 (Plerixafor and Motixafortide) are currently used for the treatment of MM patients for stem cell mobilization prior autologous transplantation (70, 71). Next, we mined ClinicalTrials.gov and examined whether any of the remaining 12 FDA-approved drugs (not specifically for MM) are undergoing or have completed clinical trials in MM patients (72). We came across 3 completed clinical trials (NCT01582295, NCT03201250 and NCT01866293) that evaluated the effectiveness of Cabozantinib, with poor results as monotherapy in relapsed/refractory MM patients. Collectively, after all these filtering steps we identified 11 FDA-approved drugs that target 4 of the 28 MM gene products (EDNRB, HGF, CCL3, ADM) and have not yet undergone evaluation in MM patients (**Table 2**). These drugs represent potentially important therapeutic options and should be given priority for future studies.

**Table 2:** list of the 11 FDA-approved drugs that inhibit 4 MM gene products.

DRUG	MECHANISM OF ACTION	TARGETED GENE	CURRENT USE	SOURCE
CAPMATINIB	HGF Receptor Inhibitor	HGF	Non-Small Cell Lung Cancer	DRUGBANK, ChEMBL, IUPHAR/BPS
TEPOTINIB	HGF Receptor Inhibitor	HGF	Non-Small Cell Lung Cancer	DRUGBANK, ChEMBL, IUPHAR/BPS
AMIVANTAMAB	HGF Receptor Inhibitor	HGF	Non-Small Cell Lung Cancer	DRUGBANK, ChEMBL
BOSENTAN	Endothelin Receptor type A/B inhibitor	EDNRB	Pulmonary Arterial Hypertension	DRUGBANK, ChEMBL, IUPHAR/BPS
MACITENTAN	Endothelin Receptor type A/B inhibitor	EDNRB	Pulmonary Arterial Hypertension	DRUGBANK, ChEMBL, IUPHAR/BPS
ATOGEPANT	CGRP Receptor Inhibitor	ADM	Migraine Prophylaxis	DRUGBANK, IUPHAR/BPS, ChEMBL
ZAVEGEPANT	CGRP Receptor Inhibitor	ADM	Migraine Treatment	DRUGBANK, IUPHAR/BPS
RIMEGEPANT	CGRP Receptor Inhibitor	ADM	Migraine Treatment	DRUGBANK, ChEMBL, IUPHAR/BPS
UBROGEPANT	CGRP Receptor Inhibitor	ADM	Migraine Treatment	DRUGBANK, ChEMBL, IUPHAR/BPS
ERENUMAB	CGRP Receptor Inhibitor	ADM	Migraine Prophylaxis	DRUGBANK, ChEMBL, IUPHAR/BPS
MARAVIROC	CCR5 Inhibitor	CCL3	HIV infection	DRUGBANK, ChEMBL, IUPHAR/BPS

**Table 2** summarizes the list of 11 FDA-approved drugs that inhibit 4 of the 28 MM gene products (HGF, EDNRB, ADM and CCL3) along with their current use. These drugs have not yet undergone evaluation in MM patients and thus represent potentially important therapeutic options that need to be further explored in the future.

## 4 Discussion

Gene expression profiling has emerged as a powerful tool which has led to a significant improvement in our understanding of MM biology. From hybridization-based assays, such as microarrays, to high throughput sequencing approaches, GEP studies have undoubtedly proven to be useful in molecular classification, patient stratification, survival prediction and treatment response prognostication (73–76). Combined with the enormous amount of transcriptomic data available in public domain, GEP studies represent a valuable tool in modern myeloma research.

To offer a more comprehensive view of transcriptomic abnormalities of NDMM patients we analyzed publicly available mRNA data of NDMM patients and HD from 7 studies. We first extracted DE genes with great significance (log2fc ≥2 and P-adj <0.05) between NDMM patients and healthy individuals from each study, separately. Subsequently, by comparing the lists of DE genes and by applying strict filtering criteria we identified 28 MM-associated genes that were consistently DE, with 22 of them being upregulated in the NDMM group. Even though the fold change and the p-values of all the 28 genes were significant, their expression measures were highly variable, with TMM values ranging from less than 1 to greater than 100. It is reasonable to assume that genes with higher expression values are more likely to be involved in MM pathogenesis. However, it is erroneous to set a threshold and filter the genes with lower values based on the assumption that they probably represent transcriptional noise rather than truly over/under-expressed genes. For example, the PTPRC gene coding for CD45, which is known for its partially positive surface expression in normal PCs and its partially heterogenous surface expression in MM PCs, was among the genes with the lowest TMM values (77–79). Additionally, due to many levels of complex gene regulation, a non-linear relationship exists between mRNA and protein abundance and function. Thus, mRNA expression measures alone are inadequate to draw safe conclusions and should be complimented by other omics and functional assays.

Reassuringly, 13 of the 28 genes that we identified are well documented to play a role in MM, thus serving as a quality control for our methods and criteria. CCND1 and DKK1 were among the genes that have a well-established relationship with MM. CCND1 encodes cyclin d1 which facilitates cell cycle progression from G0 towards S phase (80). Interestingly, even though overexpression of cyclin D1 is mostly associated with t(11;14) translocation, we observed that even patients without the aforementioned translocation had elevated expression measures of CCND1 gene implying that cyclin D1 involvement in MM exceeds t(11;14) translocation. DKK1 encodes a soluble factor which shifts the normal balance of bone remodeling in favor of resorption (81, 82). Of note, among the list of 144 experimental drugs that we extracted from drug databases, we observed 2 investigational monoclonal antibodies that neutralize DKK1 protein (BHQ-880 and DKN-01).

In addition to ‘known’ MM genes, we identified 4 genes that were not previously considered to contribute to MM biology, with 3 of them encoding long non-coding RNA molecules. Mechanistically, the lncRNA gene PURPL is of great interest since it is implied to inactivate p53 protein in colorectal cancer (40). Nevertheless, it should be stated that PURPL’s mRNA abundance was overall low compared to the other genes that have an established linkage with MM pathogenesis and therefore further functional assays are needed to characterize its impact.

Interestingly, we observed that 9 of the 28 genes were over/under-expressed by the great majority (>75%) of NDMM patients. We also examined if any gene was over/under-expressed in a subgroup-specific manner, but results were inconclusive, possibly due to the relatively small number of samples per subgroup. Additionally, we compared the 28 genes with DE genes from patients with premalignant plasma cell disorders and HD and found an overlap of 10 genes, implying that these genes might contribute to malignant transformation of normal PCs.

Since drug repurposing is a valuable cost-effective and relatively fast alternative to traditional drug discovery (83–85), we investigated whether existing drugs target any of the 22 over-expressed MM gene products. By mining three drug databases, we identified 175 drugs that target 8 of the 22 MM genes. After excluding the experimental drugs, the drugs that target MM gene molecules with an off-target MOA, the drugs that are currently used in MM treatment and the drugs that have already been evaluated for the treatment of MM in previous clinical trials, we resulted in 11 drugs that target 4 MM key genes (EDNRB, HGF, CCL3 and ADM). These drugs have already received FDA approval for the treatment of other diseases and successfully passed safety requirements in previous clinical trials. Therefore, they represent highly attractive therapeutic options and should be explored further.

Endothelin receptor (EDNR) antagonists (Bosentan and Macitentan) are currently used to treat patients with pulmonary hypertension (86). They bind EDNR type A (EDNRA) and EDNR type B (EDNRB) and inhibit endothelin 1 activity. Aberrant activation of endothelin 1 axis is implied in several malignancies, including multiple myeloma (9, 36). In MM, EDNRA is detected both in primary myeloma cells and normal PCs while EDNRB is detected only in primary MM cells (87). This previously reported restricted expression pattern of EDNRB in myeloma cells is in accordance with our findings and explains the high fold change of EDNRB gene that we identified. Preclinical studies assessing efficacy of EDNR inhibition showed promising results. Pharmaceutical EDNR blockage in MM cell lines with bosentan or macitentan decreased viability of cultured cells (88, 89). Similarly, combination of bosentan and bortezomib had stronger antiproliferative effects in myeloma cell lines than bosentan or bortezomib alone implying a synergistic effect of EDNR antagonists and proteasome inhibitors (87).

Hepatocyte growth factor receptor (HGFR) inhibitors antagonize HGF/c-MET binding. Upon binding, c-MET transduces HGF-mediated pro-survival signal by activating MAPK and PI3K/PKB signaling pathways which in turn favor MM cell proliferation (34). Interestingly, elevated serum levels of HGF correlate with poor treatment response and inferior overall survival (90). Previous clinical trials assessing HGFR inhibitors (Cabozantinib and Sunitinib) as monotherapy in relapsed/refractory MM patients failed to exhibit significant anti-tumor activity (91, 92). However, these trials enrolled patients who had advanced disease which is difficult to suppress with single-agent regimens. Our study suggests that HGF has an important role in multiple myeloma. HGF gene was up-regulated in 6 of the 7 studies whereas nearly 80% of NDMM patients over-expressed the HGF gene compared to healthy individuals. Undoubtedly, further clinical trials are needed with HGFR antagonists before this drug class is abandoned.

CC motif chemokine receptor type 1 and 5 (CCR1 and CCR5) inhibitors antagonize chemokine ligand 3 (CCL3), a proinflammatory protein belonging to the C-C chemokine family. Tumor-derived CCL3 induces MBD by affecting bone resorption and formation (93, 94). Additionally, CCL3 enhances MM-mediated anemia by suppressing erythropoiesis through GATA1 downregulation (95, 96). According to our findings, CCL3 gene was over-expressed by 50% of NDMM patients. Although upregulation of CCL3 was not a unifying molecular abnormality, patients who over-expressed CCL3 gene had abundantly high expression values underlining that CCL3 inhibition with CCR1/5 antagonists could be a potential therapeutic approach for patients whose disease complications (MBD and anemia) are driven by CCL3 (**Supplementary Figure 4**).

Lastly, Calcitonin gene-related peptide receptor (CGRPR) inhibitors antagonize the CT/CGRP family of peptides (97). Members of the CT/CGRP family of peptides include calcitonin, calcitonin gene-related peptide, amylin, intermedin and adrenomedullin (98). Adrenomedullin, encoded by ADM gene, is a peptide initially isolated from human pheochromocytoma tissues and described as a hypotensive factor (99). Since then, intensive research has revealed its versatile role in vascularization and vasodilation (100). In addition to its physiological role, adrenomedullin is implicated in the pathogenesis of several malignancies, including breast cancer and melanoma (101). In accordance with the documented role of adrenomedullin in various cancers, we observed that high expression of ADM is a common feature among NDMM patients. ADM gene was found upregulated in the NDMM group of 4 datasets with a mean log2fc value of 3.1. Previous functional assays revealed that adrenomedullin enhances MM-driven neo-angiogenesis implying that CGRPR inhibitors represent a drug class that could potentially reverse MM’s angiogenic switch (52).

In conclusion, to our knowledge this is the first study that performed a comparative analysis of publicly available gene expression datasets in order to investigate abnormal transcriptomic patterns of newly diagnosed multiple myeloma. Taken together, our study offers insights in MM pathogenesis and reveals potential new treatment options that, for starters, could be tested in pretreated MM patients with no other therapeutic options. Future studies are needed to further corroborate our findings and to evaluate the clinical significance of these drugs and their targets.

## Data availability statement

The original contributions presented in the study are included in the article/**Supplementary Material**. Further inquiries can be directed to the corresponding authors.

## Ethics statement

Ethical approval was not required for the study involving humans in accordance with the local legislation and institutional requirements. Written informed consent to participate in this study was not required from the participants or the participants’ legal guardians/next of kin in accordance with the national legislation and the institutional requirements.

## Author contributions

AG: Data curation, Formal analysis, Investigation, Methodology, Writing – original draft, Writing – review & editing. MN: Data curation, Formal analysis, Investigation, Methodology, Writing – original draft. GA: Conceptualization, Formal analysis, Methodology, Project administration, Supervision, Validation, Writing – review & editing. NG: Conceptualization, Formal analysis, Methodology, Project administration, Supervision, Validation, Writing – review & editing.
