# Supplementary material for: A comparative analysis of transcriptomics of newly diagnosed multiple myeloma: exploring drug repurposing
Source: Front Oncol. 2024 Apr 16;14:1390105. doi: 10.3389/fonc.2024.1390105 (PMC11058662; doi:10.3389/fonc.2024.1390105)
Supplement: Supplementary file 1 [file DataSheet_1.docx]

***Supplementary Material***

**1. Supplementary data:**

**1.1 Supplementary data 1.** Comparison of the 28 MM genes with differentially expressed (DE) genes between healthy donors and patients with premalignant plasma cell disorders.

To compare the 28 genes with DE genes between healthy donors and patients with premalignant plasma cell disorders, we mined DE genes between healthy donors (n=22) and MGUS patients (n=44) from GSE5900 GEO dataset, between healthy donors (n=15) and MGUS patients (n=22) from GSE6477 GEO dataset, between healthy donors (n=22) and smoldering multiple myeloma (SMM) patients (n=12) from GSE5900 GEO dataset and between healthy donors (n=15) and SMM patients (n=24) from GSE6477 GEO dataset. After mining and filtering the lists of degs, we selected positively the DE genes with absolute log2fc≥ 2 and P-adj< 0.05. Next, we compared the lists of DE genes between healthy donors and MGUS patients and between healthy donors and SMM patients from the 2 datasets and selected positively the overlapping genes. Afterwards, we compared the 28 MM genes with the 2 lists of overlapping genes. We identified an overlap of 2 genes (HGF and CCND1) between the 28 genes and the overlapping DE genes between healthy donors and MGUS patients and an overlap of 10 genes (HGF, CCND1, GADD45A, DUSP4, NDNF, BTBD3, ST3GAL6, CD81, CD27 and PTPRC) between the 28 genes and the overlapping DE genes between healthy donors and SMM patients. As mentioned in the main manuscript, 7 (HGF, CCND1, GADD45A, DUSP4, NDNF, BTBD3, ST3GAL6) and 3 (CD81, CD27 and PTPRC) genes were found upregulated and downregulated respectively in patients with premalignant plasma cell disorders, compared to healthy donors.

**2. Supplementary Figures and Tables**

**2.1 Supplementary Figures**

**
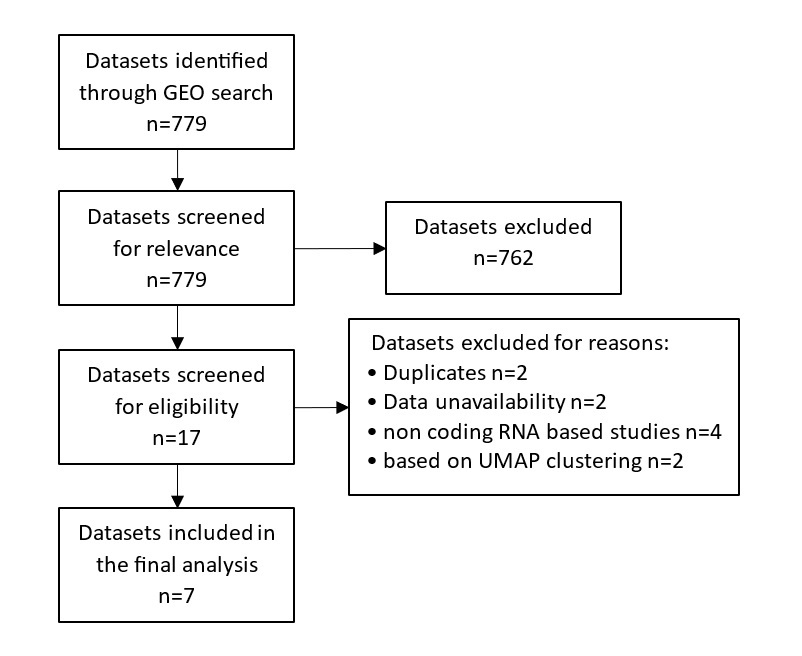
**

**Supplementary Figure 1.** Flowchart for the identification of eligible datasets.

**Supplementary Figure 2 (**Uploaded as a separate supplementary file**).** Comparison of relative expression of the 28 genes between healthy donors (HD) and newly diagnosed multiple myeloma patients (NDMM) from GSE153380 dataset and GSE175384 dataset. TMM values were log-transformed prior plotting. TMM values less than 0.1 were plotted as 0.1.

**
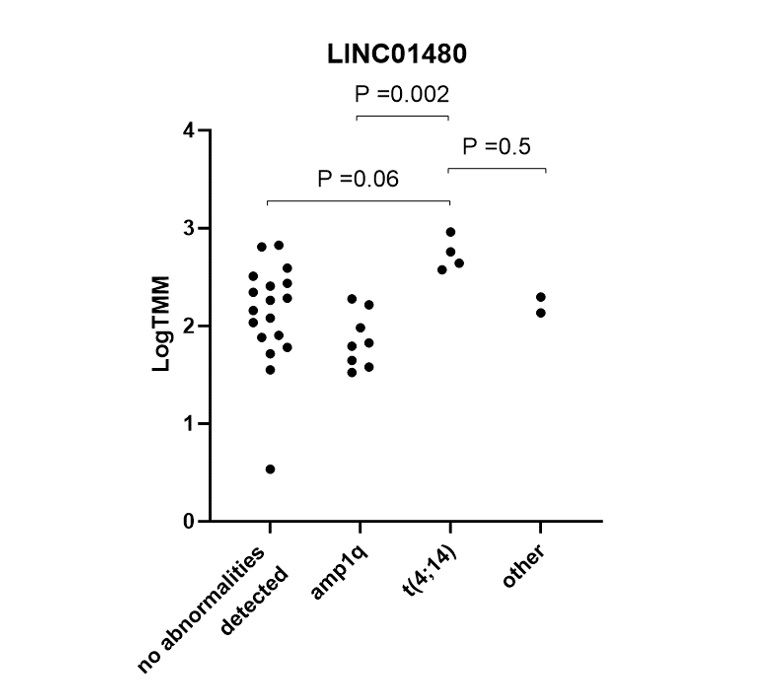
**

**Supplementary Figure 3.** Comparison of relative expression of LINC01480 gene between MM subgroups from GSE175384 dataset.

**
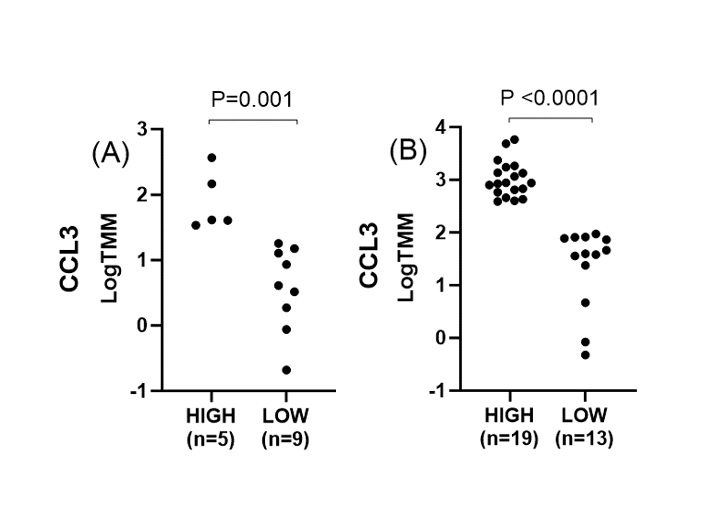
**

**Supplementary Figure 4.** Comparison of relative expression of CCL3 gene of NDMM patients from GSE153380 (A) dataset and GSE175384 (B) dataset. Patients were divided into 2 groups, those who over-expressed CCL3 gene (HIGH) and those who did not over-express CCL3 gene (LOW).

**2.2 Supplementary Tables**

| **GEO SAMPLE ID** | **SAMPLE TYPE** | **STUDY/DATASET** |
| --- | --- | --- |
| GSM5332228 | HEALTHY DONOR | GSE175384 |
| GSM5332229 | HEALTHY DONOR | GSE175384 |
| GSM5332230 | HEALTHY DONOR | GSE175384 |
| GSM5332231 | HEALTHY DONOR | GSE175384 |
| GSM5332232 | HEALTHY DONOR | GSE175384 |
| GSM5332233 | HEALTHY DONOR | GSE175384 |
| GSM5332234 | HEALTHY DONOR | GSE175384 |
| GSM5332235 | HEALTHY DONOR | GSE175384 |
| GSM5332263 | NDMM | GSE175384 |
| GSM5332264 | NDMM | GSE175384 |
| GSM5332265 | NDMM | GSE175384 |
| GSM5332266 | NDMM | GSE175384 |
| GSM5332267 | NDMM | GSE175384 |
| GSM5332268 | NDMM | GSE175384 |
| GSM5332269 | NDMM | GSE175384 |
| GSM5332270 | NDMM | GSE175384 |
| GSM5332271 | NDMM | GSE175384 |
| GSM5332272 | NDMM | GSE175384 |
| GSM5332273 | NDMM | GSE175384 |
| GSM5332274 | NDMM | GSE175384 |
| GSM5332275 | NDMM | GSE175384 |
| GSM5332276 | NDMM | GSE175384 |
| GSM5332277 | NDMM | GSE175384 |
| GSM5332278 | NDMM | GSE175384 |
| GSM5332279 | NDMM | GSE175384 |
| GSM5332280 | NDMM | GSE175384 |
| GSM5332281 | NDMM | GSE175384 |
| GSM5332282 | NDMM | GSE175384 |
| GSM5332283 | NDMM | GSE175384 |
| GSM5332284 | NDMM | GSE175384 |
| GSM5332285 | NDMM | GSE175384 |
| GSM5332286 | NDMM | GSE175384 |
| GSM5332287 | NDMM | GSE175384 |
| GSM5332288 | NDMM | GSE175384 |
| GSM5332289 | NDMM | GSE175384 |
| GSM5332290 | NDMM | GSE175384 |
| GSM5332291 | NDMM | GSE175384 |
| GSM5332292 | NDMM | GSE175384 |
| GSM5332293 | NDMM | GSE175384 |
| GSM5332294 | NDMM | GSE175384 |
| GSM4643279 | HEALTHY DONOR | GSE153380 |
| GSM4643280 | HEALTHY DONOR | GSE153380 |
| GSM4643281 | HEALTHY DONOR | GSE153380 |
| GSM4643282 | HEALTHY DONOR | GSE153380 |
| GSM4643267 | HEALTHY DONOR | GSE153380 |
| GSM4643285 | NDMM | GSE153380 |
| GSM4643275 | NDMM | GSE153380 |
| GSM4643269 | NDMM | GSE153380 |
| GSM4643284 | NDMM | GSE153380 |
| GSM4643276 | NDMM | GSE153380 |
| GSM4643259 | NDMM | GSE153380 |
| GSM4643268 | NDMM | GSE153380 |
| GSM4643290 | NDMM | GSE153380 |
| GSM4643260 | NDMM | GSE153380 |
| GSM4643273 | NDMM | GSE153380 |
| GSM4643289 | NDMM | GSE153380 |
| GSM4643274 | NDMM | GSE153380 |
| GSM4643270 | NDMM | GSE153380 |
| GSM4643277 | NDMM | GSE153380 |
| [GSM3227604](https://www.ncbi.nlm.nih.gov/geo/query/acc.cgi?acc=GSM3227604) | HEALTHY DONOR | GSE116294 |
| [GSM3227605](https://www.ncbi.nlm.nih.gov/geo/query/acc.cgi?acc=GSM3227605) | HEALTHY DONOR | GSE116294 |
| [GSM3227606](https://www.ncbi.nlm.nih.gov/geo/query/acc.cgi?acc=GSM3227606) | HEALTHY DONOR | GSE116294 |
| [GSM3227607](https://www.ncbi.nlm.nih.gov/geo/query/acc.cgi?acc=GSM3227607) | HEALTHY DONOR | GSE116294 |
| [GSM3227608](https://www.ncbi.nlm.nih.gov/geo/query/acc.cgi?acc=GSM3227608) | NDMM | GSE116294 |
| [GSM3227609](https://www.ncbi.nlm.nih.gov/geo/query/acc.cgi?acc=GSM3227609) | NDMM | GSE116294 |
| [GSM3227610](https://www.ncbi.nlm.nih.gov/geo/query/acc.cgi?acc=GSM3227610) | NDMM | GSE116294 |
| [GSM3227611](https://www.ncbi.nlm.nih.gov/geo/query/acc.cgi?acc=GSM3227611) | NDMM | GSE116294 |
| [GSM3227612](https://www.ncbi.nlm.nih.gov/geo/query/acc.cgi?acc=GSM3227612) | NDMM | GSE116294 |
| [GSM3227613](https://www.ncbi.nlm.nih.gov/geo/query/acc.cgi?acc=GSM3227613) | NDMM | GSE116294 |
| [GSM3227614](https://www.ncbi.nlm.nih.gov/geo/query/acc.cgi?acc=GSM3227614) | NDMM | GSE116294 |
| [GSM3227615](https://www.ncbi.nlm.nih.gov/geo/query/acc.cgi?acc=GSM3227615) | NDMM | GSE116294 |
| [GSM3227616](https://www.ncbi.nlm.nih.gov/geo/query/acc.cgi?acc=GSM3227616) | NDMM | GSE116294 |
| [GSM3227617](https://www.ncbi.nlm.nih.gov/geo/query/acc.cgi?acc=GSM3227617) | NDMM | GSE116294 |
| [GSM3227618](https://www.ncbi.nlm.nih.gov/geo/query/acc.cgi?acc=GSM3227618) | NDMM | GSE116294 |
| [GSM3227619](https://www.ncbi.nlm.nih.gov/geo/query/acc.cgi?acc=GSM3227619) | NDMM | GSE116294 |
| [GSM3227620](https://www.ncbi.nlm.nih.gov/geo/query/acc.cgi?acc=GSM3227620) | NDMM | GSE116294 |
| [GSM3227621](https://www.ncbi.nlm.nih.gov/geo/query/acc.cgi?acc=GSM3227621) | NDMM | GSE116294 |
| [GSM3227622](https://www.ncbi.nlm.nih.gov/geo/query/acc.cgi?acc=GSM3227622) | NDMM | GSE116294 |
| [GSM3227623](https://www.ncbi.nlm.nih.gov/geo/query/acc.cgi?acc=GSM3227623) | NDMM | GSE116294 |
| [GSM3227624](https://www.ncbi.nlm.nih.gov/geo/query/acc.cgi?acc=GSM3227624) | NDMM | GSE116294 |
| [GSM3227625](https://www.ncbi.nlm.nih.gov/geo/query/acc.cgi?acc=GSM3227625) | NDMM | GSE116294 |
| [GSM3227626](https://www.ncbi.nlm.nih.gov/geo/query/acc.cgi?acc=GSM3227626) | NDMM | GSE116294 |
| [GSM3227627](https://www.ncbi.nlm.nih.gov/geo/query/acc.cgi?acc=GSM3227627) | NDMM | GSE116294 |
| [GSM3227628](https://www.ncbi.nlm.nih.gov/geo/query/acc.cgi?acc=GSM3227628) | NDMM | GSE116294 |
| [GSM3227629](https://www.ncbi.nlm.nih.gov/geo/query/acc.cgi?acc=GSM3227629) | NDMM | GSE116294 |
| [GSM3227630](https://www.ncbi.nlm.nih.gov/geo/query/acc.cgi?acc=GSM3227630) | NDMM | GSE116294 |
| [GSM3227631](https://www.ncbi.nlm.nih.gov/geo/query/acc.cgi?acc=GSM3227631) | NDMM | GSE116294 |
| [GSM3227632](https://www.ncbi.nlm.nih.gov/geo/query/acc.cgi?acc=GSM3227632) | NDMM | GSE116294 |
| [GSM3227633](https://www.ncbi.nlm.nih.gov/geo/query/acc.cgi?acc=GSM3227633) | NDMM | GSE116294 |
| [GSM3227634](https://www.ncbi.nlm.nih.gov/geo/query/acc.cgi?acc=GSM3227634) | NDMM | GSE116294 |
| [GSM3227635](https://www.ncbi.nlm.nih.gov/geo/query/acc.cgi?acc=GSM3227635) | NDMM | GSE116294 |
| [GSM3227636](https://www.ncbi.nlm.nih.gov/geo/query/acc.cgi?acc=GSM3227636) | NDMM | GSE116294 |
| [GSM3227637](https://www.ncbi.nlm.nih.gov/geo/query/acc.cgi?acc=GSM3227637) | NDMM | GSE116294 |
| [GSM3227638](https://www.ncbi.nlm.nih.gov/geo/query/acc.cgi?acc=GSM3227638) | NDMM | GSE116294 |
| [GSM3227639](https://www.ncbi.nlm.nih.gov/geo/query/acc.cgi?acc=GSM3227639) | NDMM | GSE116294 |
| [GSM3227640](https://www.ncbi.nlm.nih.gov/geo/query/acc.cgi?acc=GSM3227640) | NDMM | GSE116294 |
| [GSM3227641](https://www.ncbi.nlm.nih.gov/geo/query/acc.cgi?acc=GSM3227641) | NDMM | GSE116294 |
| [GSM3227642](https://www.ncbi.nlm.nih.gov/geo/query/acc.cgi?acc=GSM3227642) | NDMM | GSE116294 |
| [GSM3227643](https://www.ncbi.nlm.nih.gov/geo/query/acc.cgi?acc=GSM3227643) | NDMM | GSE116294 |
| [GSM3227644](https://www.ncbi.nlm.nih.gov/geo/query/acc.cgi?acc=GSM3227644) | NDMM | GSE116294 |
| [GSM3227645](https://www.ncbi.nlm.nih.gov/geo/query/acc.cgi?acc=GSM3227645) | NDMM | GSE116294 |
| [GSM3227646](https://www.ncbi.nlm.nih.gov/geo/query/acc.cgi?acc=GSM3227646) | NDMM | GSE116294 |
| [GSM3227647](https://www.ncbi.nlm.nih.gov/geo/query/acc.cgi?acc=GSM3227647) | NDMM | GSE116294 |
| [GSM3227648](https://www.ncbi.nlm.nih.gov/geo/query/acc.cgi?acc=GSM3227648) | NDMM | GSE116294 |
| [GSM3227649](https://www.ncbi.nlm.nih.gov/geo/query/acc.cgi?acc=GSM3227649) | NDMM | GSE116294 |
| [GSM3227650](https://www.ncbi.nlm.nih.gov/geo/query/acc.cgi?acc=GSM3227650) | NDMM | GSE116294 |
| [GSM3227651](https://www.ncbi.nlm.nih.gov/geo/query/acc.cgi?acc=GSM3227651) | NDMM | GSE116294 |
| [GSM3227652](https://www.ncbi.nlm.nih.gov/geo/query/acc.cgi?acc=GSM3227652) | NDMM | GSE116294 |
| [GSM3227653](https://www.ncbi.nlm.nih.gov/geo/query/acc.cgi?acc=GSM3227653) | NDMM | GSE116294 |
| [GSM3227654](https://www.ncbi.nlm.nih.gov/geo/query/acc.cgi?acc=GSM3227654) | NDMM | GSE116294 |
| [GSM3227655](https://www.ncbi.nlm.nih.gov/geo/query/acc.cgi?acc=GSM3227655) | NDMM | GSE116294 |
| [GSM3227656](https://www.ncbi.nlm.nih.gov/geo/query/acc.cgi?acc=GSM3227656) | NDMM | GSE116294 |
| [GSM3227657](https://www.ncbi.nlm.nih.gov/geo/query/acc.cgi?acc=GSM3227657) | NDMM | GSE116294 |
| [GSM154434](https://www.ncbi.nlm.nih.gov/geo/query/acc.cgi?acc=GSM154434) | HEALTHY DONOR | GSE6691 |
| [GSM154436](https://www.ncbi.nlm.nih.gov/geo/query/acc.cgi?acc=GSM154436) | HEALTHY DONOR | GSE6691 |
| [GSM154437](https://www.ncbi.nlm.nih.gov/geo/query/acc.cgi?acc=GSM154437) | HEALTHY DONOR | GSE6691 |
| [GSM154438](https://www.ncbi.nlm.nih.gov/geo/query/acc.cgi?acc=GSM154438) | HEALTHY DONOR | GSE6691 |
| [GSM154439](https://www.ncbi.nlm.nih.gov/geo/query/acc.cgi?acc=GSM154439) | HEALTHY DONOR | GSE6691 |
| [GSM154217](https://www.ncbi.nlm.nih.gov/geo/query/acc.cgi?acc=GSM154217) | NDMM | GSE6691 |
| [GSM154219](https://www.ncbi.nlm.nih.gov/geo/query/acc.cgi?acc=GSM154219) | NDMM | GSE6691 |
| [GSM154220](https://www.ncbi.nlm.nih.gov/geo/query/acc.cgi?acc=GSM154220) | NDMM | GSE6691 |
| [GSM154221](https://www.ncbi.nlm.nih.gov/geo/query/acc.cgi?acc=GSM154221) | NDMM | GSE6691 |
| [GSM154223](https://www.ncbi.nlm.nih.gov/geo/query/acc.cgi?acc=GSM154223) | NDMM | GSE6691 |
| [GSM154224](https://www.ncbi.nlm.nih.gov/geo/query/acc.cgi?acc=GSM154224) | NDMM | GSE6691 |
| [GSM154225](https://www.ncbi.nlm.nih.gov/geo/query/acc.cgi?acc=GSM154225) | NDMM | GSE6691 |
| [GSM154227](https://www.ncbi.nlm.nih.gov/geo/query/acc.cgi?acc=GSM154227) | NDMM | GSE6691 |
| [GSM154228](https://www.ncbi.nlm.nih.gov/geo/query/acc.cgi?acc=GSM154228) | NDMM | GSE6691 |
| [GSM154229](https://www.ncbi.nlm.nih.gov/geo/query/acc.cgi?acc=GSM154229) | NDMM | GSE6691 |
| [GSM154231](https://www.ncbi.nlm.nih.gov/geo/query/acc.cgi?acc=GSM154231) | NDMM | GSE6691 |
| [GSM154232](https://www.ncbi.nlm.nih.gov/geo/query/acc.cgi?acc=GSM154232) | NDMM | GSE6691 |
| [GSM1152350](https://www.ncbi.nlm.nih.gov/geo/query/acc.cgi?acc=GSM1152350) | HEALTHY DONOR | GSE47552 |
| [GSM1152351](https://www.ncbi.nlm.nih.gov/geo/query/acc.cgi?acc=GSM1152351) | HEALTHY DONOR | GSE47552 |
| [GSM1152352](https://www.ncbi.nlm.nih.gov/geo/query/acc.cgi?acc=GSM1152352) | HEALTHY DONOR | GSE47552 |
| [GSM1152353](https://www.ncbi.nlm.nih.gov/geo/query/acc.cgi?acc=GSM1152353) | HEALTHY DONOR | GSE47552 |
| [GSM1152354](https://www.ncbi.nlm.nih.gov/geo/query/acc.cgi?acc=GSM1152354) | HEALTHY DONOR | GSE47552 |
| [GSM1152309](https://www.ncbi.nlm.nih.gov/geo/query/acc.cgi?acc=GSM1152309) | NDMM | GSE47552 |
| [GSM1152310](https://www.ncbi.nlm.nih.gov/geo/query/acc.cgi?acc=GSM1152310) | NDMM | GSE47552 |
| [GSM1152311](https://www.ncbi.nlm.nih.gov/geo/query/acc.cgi?acc=GSM1152311) | NDMM | GSE47552 |
| [GSM1152312](https://www.ncbi.nlm.nih.gov/geo/query/acc.cgi?acc=GSM1152312) | NDMM | GSE47552 |
| [GSM1152313](https://www.ncbi.nlm.nih.gov/geo/query/acc.cgi?acc=GSM1152313) | NDMM | GSE47552 |
| [GSM1152314](https://www.ncbi.nlm.nih.gov/geo/query/acc.cgi?acc=GSM1152314) | NDMM | GSE47552 |
| [GSM1152315](https://www.ncbi.nlm.nih.gov/geo/query/acc.cgi?acc=GSM1152315) | NDMM | GSE47552 |
| [GSM1152316](https://www.ncbi.nlm.nih.gov/geo/query/acc.cgi?acc=GSM1152316) | NDMM | GSE47552 |
| [GSM1152317](https://www.ncbi.nlm.nih.gov/geo/query/acc.cgi?acc=GSM1152317) | NDMM | GSE47552 |
| [GSM1152318](https://www.ncbi.nlm.nih.gov/geo/query/acc.cgi?acc=GSM1152318) | NDMM | GSE47552 |
| [GSM1152319](https://www.ncbi.nlm.nih.gov/geo/query/acc.cgi?acc=GSM1152319) | NDMM | GSE47552 |
| [GSM1152320](https://www.ncbi.nlm.nih.gov/geo/query/acc.cgi?acc=GSM1152320) | NDMM | GSE47552 |
| [GSM1152321](https://www.ncbi.nlm.nih.gov/geo/query/acc.cgi?acc=GSM1152321) | NDMM | GSE47552 |
| [GSM1152322](https://www.ncbi.nlm.nih.gov/geo/query/acc.cgi?acc=GSM1152322) | NDMM | GSE47552 |
| [GSM1152323](https://www.ncbi.nlm.nih.gov/geo/query/acc.cgi?acc=GSM1152323) | NDMM | GSE47552 |
| [GSM1152324](https://www.ncbi.nlm.nih.gov/geo/query/acc.cgi?acc=GSM1152324) | NDMM | GSE47552 |
| [GSM1152325](https://www.ncbi.nlm.nih.gov/geo/query/acc.cgi?acc=GSM1152325) | NDMM | GSE47552 |
| [GSM1152326](https://www.ncbi.nlm.nih.gov/geo/query/acc.cgi?acc=GSM1152326) | NDMM | GSE47552 |
| [GSM1152327](https://www.ncbi.nlm.nih.gov/geo/query/acc.cgi?acc=GSM1152327) | NDMM | GSE47552 |
| [GSM1152328](https://www.ncbi.nlm.nih.gov/geo/query/acc.cgi?acc=GSM1152328) | NDMM | GSE47552 |
| [GSM1152329](https://www.ncbi.nlm.nih.gov/geo/query/acc.cgi?acc=GSM1152329) | NDMM | GSE47552 |
| [GSM1152330](https://www.ncbi.nlm.nih.gov/geo/query/acc.cgi?acc=GSM1152330) | NDMM | GSE47552 |
| [GSM1152331](https://www.ncbi.nlm.nih.gov/geo/query/acc.cgi?acc=GSM1152331) | NDMM | GSE47552 |
| [GSM1152332](https://www.ncbi.nlm.nih.gov/geo/query/acc.cgi?acc=GSM1152332) | NDMM | GSE47552 |
| [GSM1152333](https://www.ncbi.nlm.nih.gov/geo/query/acc.cgi?acc=GSM1152333) | NDMM | GSE47552 |
| [GSM1152334](https://www.ncbi.nlm.nih.gov/geo/query/acc.cgi?acc=GSM1152334) | NDMM | GSE47552 |
| [GSM1152335](https://www.ncbi.nlm.nih.gov/geo/query/acc.cgi?acc=GSM1152335) | NDMM | GSE47552 |
| [GSM1152336](https://www.ncbi.nlm.nih.gov/geo/query/acc.cgi?acc=GSM1152336) | NDMM | GSE47552 |
| [GSM1152337](https://www.ncbi.nlm.nih.gov/geo/query/acc.cgi?acc=GSM1152337) | NDMM | GSE47552 |
| [GSM1152338](https://www.ncbi.nlm.nih.gov/geo/query/acc.cgi?acc=GSM1152338) | NDMM | GSE47552 |
| [GSM1152339](https://www.ncbi.nlm.nih.gov/geo/query/acc.cgi?acc=GSM1152339) | NDMM | GSE47552 |
| [GSM1152340](https://www.ncbi.nlm.nih.gov/geo/query/acc.cgi?acc=GSM1152340) | NDMM | GSE47552 |
| [GSM1152341](https://www.ncbi.nlm.nih.gov/geo/query/acc.cgi?acc=GSM1152341) | NDMM | GSE47552 |
| [GSM1152342](https://www.ncbi.nlm.nih.gov/geo/query/acc.cgi?acc=GSM1152342) | NDMM | GSE47552 |
| [GSM1152343](https://www.ncbi.nlm.nih.gov/geo/query/acc.cgi?acc=GSM1152343) | NDMM | GSE47552 |
| [GSM1152344](https://www.ncbi.nlm.nih.gov/geo/query/acc.cgi?acc=GSM1152344) | NDMM | GSE47552 |
| [GSM1152345](https://www.ncbi.nlm.nih.gov/geo/query/acc.cgi?acc=GSM1152345) | NDMM | GSE47552 |
| [GSM1152346](https://www.ncbi.nlm.nih.gov/geo/query/acc.cgi?acc=GSM1152346) | NDMM | GSE47552 |
| [GSM1152347](https://www.ncbi.nlm.nih.gov/geo/query/acc.cgi?acc=GSM1152347) | NDMM | GSE47552 |
| [GSM1152348](https://www.ncbi.nlm.nih.gov/geo/query/acc.cgi?acc=GSM1152348) | NDMM | GSE47552 |
| [GSM1152349](https://www.ncbi.nlm.nih.gov/geo/query/acc.cgi?acc=GSM1152349) | NDMM | GSE47552 |
| [GSM149062](https://www.ncbi.nlm.nih.gov/geo/query/acc.cgi?acc=GSM149062) | HEALTHY DONOR | GSE6477 |
| [GSM149063](https://www.ncbi.nlm.nih.gov/geo/query/acc.cgi?acc=GSM149063) | HEALTHY DONOR | GSE6477 |
| [GSM149064](https://www.ncbi.nlm.nih.gov/geo/query/acc.cgi?acc=GSM149064) | HEALTHY DONOR | GSE6477 |
| [GSM149065](https://www.ncbi.nlm.nih.gov/geo/query/acc.cgi?acc=GSM149065) | HEALTHY DONOR | GSE6477 |
| [GSM149066](https://www.ncbi.nlm.nih.gov/geo/query/acc.cgi?acc=GSM149066) | HEALTHY DONOR | GSE6477 |
| [GSM149067](https://www.ncbi.nlm.nih.gov/geo/query/acc.cgi?acc=GSM149067) | HEALTHY DONOR | GSE6477 |
| [GSM149068](https://www.ncbi.nlm.nih.gov/geo/query/acc.cgi?acc=GSM149068) | HEALTHY DONOR | GSE6477 |
| [GSM149069](https://www.ncbi.nlm.nih.gov/geo/query/acc.cgi?acc=GSM149069) | HEALTHY DONOR | GSE6477 |
| [GSM149070](https://www.ncbi.nlm.nih.gov/geo/query/acc.cgi?acc=GSM149070) | HEALTHY DONOR | GSE6477 |
| [GSM149071](https://www.ncbi.nlm.nih.gov/geo/query/acc.cgi?acc=GSM149071) | HEALTHY DONOR | GSE6477 |
| [GSM149072](https://www.ncbi.nlm.nih.gov/geo/query/acc.cgi?acc=GSM149072) | HEALTHY DONOR | GSE6477 |
| [GSM149073](https://www.ncbi.nlm.nih.gov/geo/query/acc.cgi?acc=GSM149073) | HEALTHY DONOR | GSE6477 |
| [GSM148914](https://www.ncbi.nlm.nih.gov/geo/query/acc.cgi?acc=GSM148914) | NDMM | GSE6477 |
| [GSM148915](https://www.ncbi.nlm.nih.gov/geo/query/acc.cgi?acc=GSM148915) | NDMM | GSE6477 |
| [GSM148917](https://www.ncbi.nlm.nih.gov/geo/query/acc.cgi?acc=GSM148917) | NDMM | GSE6477 |
| [GSM148918](https://www.ncbi.nlm.nih.gov/geo/query/acc.cgi?acc=GSM148918) | NDMM | GSE6477 |
| [GSM148921](https://www.ncbi.nlm.nih.gov/geo/query/acc.cgi?acc=GSM148921) | NDMM | GSE6477 |
| [GSM148922](https://www.ncbi.nlm.nih.gov/geo/query/acc.cgi?acc=GSM148922) | NDMM | GSE6477 |
| [GSM148923](https://www.ncbi.nlm.nih.gov/geo/query/acc.cgi?acc=GSM148923) | NDMM | GSE6477 |
| [GSM148925](https://www.ncbi.nlm.nih.gov/geo/query/acc.cgi?acc=GSM148925) | NDMM | GSE6477 |
| [GSM148938](https://www.ncbi.nlm.nih.gov/geo/query/acc.cgi?acc=GSM148938) | NDMM | GSE6477 |
| [GSM148939](https://www.ncbi.nlm.nih.gov/geo/query/acc.cgi?acc=GSM148939) | NDMM | GSE6477 |
| [GSM148940](https://www.ncbi.nlm.nih.gov/geo/query/acc.cgi?acc=GSM148940) | NDMM | GSE6477 |
| [GSM148942](https://www.ncbi.nlm.nih.gov/geo/query/acc.cgi?acc=GSM148942) | NDMM | GSE6477 |
| [GSM148944](https://www.ncbi.nlm.nih.gov/geo/query/acc.cgi?acc=GSM148944) | NDMM | GSE6477 |
| [GSM148946](https://www.ncbi.nlm.nih.gov/geo/query/acc.cgi?acc=GSM148946) | NDMM | GSE6477 |
| [GSM148947](https://www.ncbi.nlm.nih.gov/geo/query/acc.cgi?acc=GSM148947) | NDMM | GSE6477 |
| [GSM148949](https://www.ncbi.nlm.nih.gov/geo/query/acc.cgi?acc=GSM148949) | NDMM | GSE6477 |
| [GSM148950](https://www.ncbi.nlm.nih.gov/geo/query/acc.cgi?acc=GSM148950) | NDMM | GSE6477 |
| [GSM148951](https://www.ncbi.nlm.nih.gov/geo/query/acc.cgi?acc=GSM148951) | NDMM | GSE6477 |
| [GSM148955](https://www.ncbi.nlm.nih.gov/geo/query/acc.cgi?acc=GSM148955) | NDMM | GSE6477 |
| [GSM148956](https://www.ncbi.nlm.nih.gov/geo/query/acc.cgi?acc=GSM148956) | NDMM | GSE6477 |
| [GSM148958](https://www.ncbi.nlm.nih.gov/geo/query/acc.cgi?acc=GSM148958) | NDMM | GSE6477 |
| [GSM148960](https://www.ncbi.nlm.nih.gov/geo/query/acc.cgi?acc=GSM148960) | NDMM | GSE6477 |
| [GSM148961](https://www.ncbi.nlm.nih.gov/geo/query/acc.cgi?acc=GSM148961) | NDMM | GSE6477 |
| [GSM148964](https://www.ncbi.nlm.nih.gov/geo/query/acc.cgi?acc=GSM148964) | NDMM | GSE6477 |
| [GSM148967](https://www.ncbi.nlm.nih.gov/geo/query/acc.cgi?acc=GSM148967) | NDMM | GSE6477 |
| [GSM148968](https://www.ncbi.nlm.nih.gov/geo/query/acc.cgi?acc=GSM148968) | NDMM | GSE6477 |
| [GSM148971](https://www.ncbi.nlm.nih.gov/geo/query/acc.cgi?acc=GSM148971) | NDMM | GSE6477 |
| [GSM148973](https://www.ncbi.nlm.nih.gov/geo/query/acc.cgi?acc=GSM148973) | NDMM | GSE6477 |
| [GSM148974](https://www.ncbi.nlm.nih.gov/geo/query/acc.cgi?acc=GSM148974) | NDMM | GSE6477 |
| [GSM148978](https://www.ncbi.nlm.nih.gov/geo/query/acc.cgi?acc=GSM148978) | NDMM | GSE6477 |
| [GSM148979](https://www.ncbi.nlm.nih.gov/geo/query/acc.cgi?acc=GSM148979) | NDMM | GSE6477 |
| [GSM148981](https://www.ncbi.nlm.nih.gov/geo/query/acc.cgi?acc=GSM148981) | NDMM | GSE6477 |
| [GSM148983](https://www.ncbi.nlm.nih.gov/geo/query/acc.cgi?acc=GSM148983) | NDMM | GSE6477 |
| [GSM148986](https://www.ncbi.nlm.nih.gov/geo/query/acc.cgi?acc=GSM148986) | NDMM | GSE6477 |
| [GSM148987](https://www.ncbi.nlm.nih.gov/geo/query/acc.cgi?acc=GSM148987) | NDMM | GSE6477 |
| [GSM148988](https://www.ncbi.nlm.nih.gov/geo/query/acc.cgi?acc=GSM148988) | NDMM | GSE6477 |
| [GSM148989](https://www.ncbi.nlm.nih.gov/geo/query/acc.cgi?acc=GSM148989) | NDMM | GSE6477 |
| [GSM148990](https://www.ncbi.nlm.nih.gov/geo/query/acc.cgi?acc=GSM148990) | NDMM | GSE6477 |
| [GSM148991](https://www.ncbi.nlm.nih.gov/geo/query/acc.cgi?acc=GSM148991) | NDMM | GSE6477 |
| [GSM148993](https://www.ncbi.nlm.nih.gov/geo/query/acc.cgi?acc=GSM148993) | NDMM | GSE6477 |
| [GSM148995](https://www.ncbi.nlm.nih.gov/geo/query/acc.cgi?acc=GSM148995) | NDMM | GSE6477 |
| [GSM148997](https://www.ncbi.nlm.nih.gov/geo/query/acc.cgi?acc=GSM148997) | NDMM | GSE6477 |
| [GSM148998](https://www.ncbi.nlm.nih.gov/geo/query/acc.cgi?acc=GSM148998) | NDMM | GSE6477 |
| [GSM148999](https://www.ncbi.nlm.nih.gov/geo/query/acc.cgi?acc=GSM148999) | NDMM | GSE6477 |
| [GSM149002](https://www.ncbi.nlm.nih.gov/geo/query/acc.cgi?acc=GSM149002) | NDMM | GSE6477 |
| [GSM149004](https://www.ncbi.nlm.nih.gov/geo/query/acc.cgi?acc=GSM149004) | NDMM | GSE6477 |
| [GSM149005](https://www.ncbi.nlm.nih.gov/geo/query/acc.cgi?acc=GSM149005) | NDMM | GSE6477 |
| [GSM149006](https://www.ncbi.nlm.nih.gov/geo/query/acc.cgi?acc=GSM149006) | NDMM | GSE6477 |
| [GSM149007](https://www.ncbi.nlm.nih.gov/geo/query/acc.cgi?acc=GSM149007) | NDMM | GSE6477 |
| [GSM149008](https://www.ncbi.nlm.nih.gov/geo/query/acc.cgi?acc=GSM149008) | NDMM | GSE6477 |
| [GSM149009](https://www.ncbi.nlm.nih.gov/geo/query/acc.cgi?acc=GSM149009) | NDMM | GSE6477 |
| [GSM149010](https://www.ncbi.nlm.nih.gov/geo/query/acc.cgi?acc=GSM149010) | NDMM | GSE6477 |
| [GSM149016](https://www.ncbi.nlm.nih.gov/geo/query/acc.cgi?acc=GSM149016) | NDMM | GSE6477 |
| [GSM149017](https://www.ncbi.nlm.nih.gov/geo/query/acc.cgi?acc=GSM149017) | NDMM | GSE6477 |
| [GSM149018](https://www.ncbi.nlm.nih.gov/geo/query/acc.cgi?acc=GSM149018) | NDMM | GSE6477 |
| [GSM149019](https://www.ncbi.nlm.nih.gov/geo/query/acc.cgi?acc=GSM149019) | NDMM | GSE6477 |
| [GSM149021](https://www.ncbi.nlm.nih.gov/geo/query/acc.cgi?acc=GSM149021) | NDMM | GSE6477 |
| [GSM149025](https://www.ncbi.nlm.nih.gov/geo/query/acc.cgi?acc=GSM149025) | NDMM | GSE6477 |
| [GSM149031](https://www.ncbi.nlm.nih.gov/geo/query/acc.cgi?acc=GSM149031) | NDMM | GSE6477 |
| [GSM149033](https://www.ncbi.nlm.nih.gov/geo/query/acc.cgi?acc=GSM149033) | NDMM | GSE6477 |
| [GSM149037](https://www.ncbi.nlm.nih.gov/geo/query/acc.cgi?acc=GSM149037) | NDMM | GSE6477 |
| [GSM149038](https://www.ncbi.nlm.nih.gov/geo/query/acc.cgi?acc=GSM149038) | NDMM | GSE6477 |
| [GSM149040](https://www.ncbi.nlm.nih.gov/geo/query/acc.cgi?acc=GSM149040) | NDMM | GSE6477 |
| [GSM149044](https://www.ncbi.nlm.nih.gov/geo/query/acc.cgi?acc=GSM149044) | NDMM | GSE6477 |
| [GSM149048](https://www.ncbi.nlm.nih.gov/geo/query/acc.cgi?acc=GSM149048) | NDMM | GSE6477 |
| [GSM149049](https://www.ncbi.nlm.nih.gov/geo/query/acc.cgi?acc=GSM149049) | NDMM | GSE6477 |
| [GSM149050](https://www.ncbi.nlm.nih.gov/geo/query/acc.cgi?acc=GSM149050) | NDMM | GSE6477 |
| [GSM149052](https://www.ncbi.nlm.nih.gov/geo/query/acc.cgi?acc=GSM149052) | NDMM | GSE6477 |
| [GSM149054](https://www.ncbi.nlm.nih.gov/geo/query/acc.cgi?acc=GSM149054) | NDMM | GSE6477 |
| [GSM149055](https://www.ncbi.nlm.nih.gov/geo/query/acc.cgi?acc=GSM149055) | NDMM | GSE6477 |
| [GSM149056](https://www.ncbi.nlm.nih.gov/geo/query/acc.cgi?acc=GSM149056) | NDMM | GSE6477 |
| [GSM149059](https://www.ncbi.nlm.nih.gov/geo/query/acc.cgi?acc=GSM149059) | NDMM | GSE6477 |
| [GSM149061](https://www.ncbi.nlm.nih.gov/geo/query/acc.cgi?acc=GSM149061) | NDMM | GSE6477 |
| [GSM411222](https://www.ncbi.nlm.nih.gov/geo/query/acc.cgi?acc=GSM411222) | HEALTHY DONOR | GSE16558 |
| [GSM411231](https://www.ncbi.nlm.nih.gov/geo/query/acc.cgi?acc=GSM411231) | HEALTHY DONOR | GSE16558 |
| [GSM411232](https://www.ncbi.nlm.nih.gov/geo/query/acc.cgi?acc=GSM411232) | HEALTHY DONOR | GSE16558 |
| [GSM411233](https://www.ncbi.nlm.nih.gov/geo/query/acc.cgi?acc=GSM411233) | HEALTHY DONOR | GSE16558 |
| [GSM411234](https://www.ncbi.nlm.nih.gov/geo/query/acc.cgi?acc=GSM411234) | HEALTHY DONOR | GSE16558 |
| [GSM409113](https://www.ncbi.nlm.nih.gov/geo/query/acc.cgi?acc=GSM409113) | NDMM | GSE16558 |
| [GSM409114](https://www.ncbi.nlm.nih.gov/geo/query/acc.cgi?acc=GSM409114) | NDMM | GSE16558 |
| [GSM409115](https://www.ncbi.nlm.nih.gov/geo/query/acc.cgi?acc=GSM409115) | NDMM | GSE16558 |
| [GSM409116](https://www.ncbi.nlm.nih.gov/geo/query/acc.cgi?acc=GSM409116) | NDMM | GSE16558 |
| [GSM409117](https://www.ncbi.nlm.nih.gov/geo/query/acc.cgi?acc=GSM409117) | NDMM | GSE16558 |
| [GSM409118](https://www.ncbi.nlm.nih.gov/geo/query/acc.cgi?acc=GSM409118) | NDMM | GSE16558 |
| [GSM409119](https://www.ncbi.nlm.nih.gov/geo/query/acc.cgi?acc=GSM409119) | NDMM | GSE16558 |
| [GSM409120](https://www.ncbi.nlm.nih.gov/geo/query/acc.cgi?acc=GSM409120) | NDMM | GSE16558 |
| [GSM409121](https://www.ncbi.nlm.nih.gov/geo/query/acc.cgi?acc=GSM409121) | NDMM | GSE16558 |
| [GSM409122](https://www.ncbi.nlm.nih.gov/geo/query/acc.cgi?acc=GSM409122) | NDMM | GSE16558 |
| [GSM409123](https://www.ncbi.nlm.nih.gov/geo/query/acc.cgi?acc=GSM409123) | NDMM | GSE16558 |
| [GSM409125](https://www.ncbi.nlm.nih.gov/geo/query/acc.cgi?acc=GSM409125) | NDMM | GSE16558 |
| [GSM409655](https://www.ncbi.nlm.nih.gov/geo/query/acc.cgi?acc=GSM409655) | NDMM | GSE16558 |
| [GSM409660](https://www.ncbi.nlm.nih.gov/geo/query/acc.cgi?acc=GSM409660) | NDMM | GSE16558 |
| [GSM409661](https://www.ncbi.nlm.nih.gov/geo/query/acc.cgi?acc=GSM409661) | NDMM | GSE16558 |
| [GSM409662](https://www.ncbi.nlm.nih.gov/geo/query/acc.cgi?acc=GSM409662) | NDMM | GSE16558 |
| [GSM409663](https://www.ncbi.nlm.nih.gov/geo/query/acc.cgi?acc=GSM409663) | NDMM | GSE16558 |
| [GSM409664](https://www.ncbi.nlm.nih.gov/geo/query/acc.cgi?acc=GSM409664) | NDMM | GSE16558 |
| [GSM409665](https://www.ncbi.nlm.nih.gov/geo/query/acc.cgi?acc=GSM409665) | NDMM | GSE16558 |
| [GSM409666](https://www.ncbi.nlm.nih.gov/geo/query/acc.cgi?acc=GSM409666) | NDMM | GSE16558 |
| [GSM409667](https://www.ncbi.nlm.nih.gov/geo/query/acc.cgi?acc=GSM409667) | NDMM | GSE16558 |
| [GSM409668](https://www.ncbi.nlm.nih.gov/geo/query/acc.cgi?acc=GSM409668) | NDMM | GSE16558 |
| [GSM409669](https://www.ncbi.nlm.nih.gov/geo/query/acc.cgi?acc=GSM409669) | NDMM | GSE16558 |
| [GSM409670](https://www.ncbi.nlm.nih.gov/geo/query/acc.cgi?acc=GSM409670) | NDMM | GSE16558 |
| [GSM410166](https://www.ncbi.nlm.nih.gov/geo/query/acc.cgi?acc=GSM410166) | NDMM | GSE16558 |
| [GSM410167](https://www.ncbi.nlm.nih.gov/geo/query/acc.cgi?acc=GSM410167) | NDMM | GSE16558 |
| [GSM410168](https://www.ncbi.nlm.nih.gov/geo/query/acc.cgi?acc=GSM410168) | NDMM | GSE16558 |
| [GSM410169](https://www.ncbi.nlm.nih.gov/geo/query/acc.cgi?acc=GSM410169) | NDMM | GSE16558 |
| [GSM410170](https://www.ncbi.nlm.nih.gov/geo/query/acc.cgi?acc=GSM410170) | NDMM | GSE16558 |
| [GSM410171](https://www.ncbi.nlm.nih.gov/geo/query/acc.cgi?acc=GSM410171) | NDMM | GSE16558 |
| [GSM410172](https://www.ncbi.nlm.nih.gov/geo/query/acc.cgi?acc=GSM410172) | NDMM | GSE16558 |
| [GSM410173](https://www.ncbi.nlm.nih.gov/geo/query/acc.cgi?acc=GSM410173) | NDMM | GSE16558 |
| [GSM410174](https://www.ncbi.nlm.nih.gov/geo/query/acc.cgi?acc=GSM410174) | NDMM | GSE16558 |
| [GSM410175](https://www.ncbi.nlm.nih.gov/geo/query/acc.cgi?acc=GSM410175) | NDMM | GSE16558 |
| [GSM410176](https://www.ncbi.nlm.nih.gov/geo/query/acc.cgi?acc=GSM410176) | NDMM | GSE16558 |
| [GSM410177](https://www.ncbi.nlm.nih.gov/geo/query/acc.cgi?acc=GSM410177) | NDMM | GSE16558 |
| [GSM410178](https://www.ncbi.nlm.nih.gov/geo/query/acc.cgi?acc=GSM410178) | NDMM | GSE16558 |
| [GSM410179](https://www.ncbi.nlm.nih.gov/geo/query/acc.cgi?acc=GSM410179) | NDMM | GSE16558 |
| [GSM410180](https://www.ncbi.nlm.nih.gov/geo/query/acc.cgi?acc=GSM410180) | NDMM | GSE16558 |
| [GSM410181](https://www.ncbi.nlm.nih.gov/geo/query/acc.cgi?acc=GSM410181) | NDMM | GSE16558 |
| [GSM410182](https://www.ncbi.nlm.nih.gov/geo/query/acc.cgi?acc=GSM410182) | NDMM | GSE16558 |
| [GSM410183](https://www.ncbi.nlm.nih.gov/geo/query/acc.cgi?acc=GSM410183) | NDMM | GSE16558 |
| [GSM410184](https://www.ncbi.nlm.nih.gov/geo/query/acc.cgi?acc=GSM410184) | NDMM | GSE16558 |
| [GSM410185](https://www.ncbi.nlm.nih.gov/geo/query/acc.cgi?acc=GSM410185) | NDMM | GSE16558 |
| [GSM410186](https://www.ncbi.nlm.nih.gov/geo/query/acc.cgi?acc=GSM410186) | NDMM | GSE16558 |
| [GSM411148](https://www.ncbi.nlm.nih.gov/geo/query/acc.cgi?acc=GSM411148) | NDMM | GSE16558 |
| [GSM411149](https://www.ncbi.nlm.nih.gov/geo/query/acc.cgi?acc=GSM411149) | NDMM | GSE16558 |
| [GSM411172](https://www.ncbi.nlm.nih.gov/geo/query/acc.cgi?acc=GSM411172) | NDMM | GSE16558 |
| [GSM411173](https://www.ncbi.nlm.nih.gov/geo/query/acc.cgi?acc=GSM411173) | NDMM | GSE16558 |
| [GSM411174](https://www.ncbi.nlm.nih.gov/geo/query/acc.cgi?acc=GSM411174) | NDMM | GSE16558 |
| [GSM411175](https://www.ncbi.nlm.nih.gov/geo/query/acc.cgi?acc=GSM411175) | NDMM | GSE16558 |
| [GSM411176](https://www.ncbi.nlm.nih.gov/geo/query/acc.cgi?acc=GSM411176) | NDMM | GSE16558 |
| [GSM411177](https://www.ncbi.nlm.nih.gov/geo/query/acc.cgi?acc=GSM411177) | NDMM | GSE16558 |
| [GSM411178](https://www.ncbi.nlm.nih.gov/geo/query/acc.cgi?acc=GSM411178) | NDMM | GSE16558 |
| [GSM411179](https://www.ncbi.nlm.nih.gov/geo/query/acc.cgi?acc=GSM411179) | NDMM | GSE16558 |
| [GSM411180](https://www.ncbi.nlm.nih.gov/geo/query/acc.cgi?acc=GSM411180) | NDMM | GSE16558 |
| [GSM411181](https://www.ncbi.nlm.nih.gov/geo/query/acc.cgi?acc=GSM411181) | NDMM | GSE16558 |
| [GSM411182](https://www.ncbi.nlm.nih.gov/geo/query/acc.cgi?acc=GSM411182) | NDMM | GSE16558 |
| [GSM411221](https://www.ncbi.nlm.nih.gov/geo/query/acc.cgi?acc=GSM411221) | NDMM | GSE16558 |

**Supplementary Table 1.** List of samples that were processed during this study, presented with their GEO accession codes.

| **GSE16558** | |  | **GSE153380** | |
| --- | --- | --- | --- | --- |
| Cytogenic abnormalities | **Number of patients** |  | **Cytogenic abnormalities** | **Number of patients** |
| no abnormalities | 12 |  | hyperdiploid | 7 |
| t(11;14) | 11 |  | t(11;14) | 2 |
| t(4;14) | 17 |  | t(4;14) | 2 |
| t(14;16) | 4 |  | t(14;16) | 1 |
| Rb deletion | 14 |  | del17p | 1 |
| Rb deletion and tp53 deletion | 1 |  | unknown status | 1 |
|  |  |  |  |  |
| **GSE116294** | |  | **GSE175384** | |
| Cytogenic abnormalities | **Number of patients** |  | **Cytogenic abnormalities** | **Number of patients** |
| Hyperdiploid | 14 |  | no abnormalities | 18 |
| t(4;14) | 12 |  | t(4;14) | 4 |
| t(14;16) | 5 |  | amp 1q | 8 |
| t(11;14) | 12 |  | del 1p | 1 |
| no alterations | 7 |  | del 1p, del17p, amp1q | 1 |
|  |  |  |  |  |
| **GSE6477** | |  | **GSE47552** | |
| **Cytogenic abnormalities** | **Number of patients** |  | **Cytogenic abnormalities** | **Number of patients** |
| hyperdiploid | 41 |  | IgH translocations | 13 |
| non-hyperdiploid | 31 |  | Non-IgH translocations | 28 |
| Unknown status | 1 |  |  |  |
|  |  |  |  |  |
| **GSE6691** | |  |  |  |
| Cytogenic abnormalities | **Number of patients** |  |  |  |
| Unknown status | 12 |  |  |  |
|  |  |  |  |  |

**Supplementary Table 2.** Cytogenetic abnormalities of each NDMM cohort.

| **GENE NAME** | **GSE153380** | **GSE175384** | **GSE116294** | **GSE6691** | **GSE47552** | **GSE6477** | **GSE16558** |
| --- | --- | --- | --- | --- | --- | --- | --- |
| CD27 | -1 | -1 | -1 | -1 | -1 | -1 | -1 |
| IGHD | 0 | 0 | -1 | -1 | 0 | -1 | 0 |
| IGHM | -1 | -1 | -1 | -1 | 0 | -1 | 0 |
| GADD45A | 1 | 1 | 1 | 0 | 1 | 0 | 1 |
| TMEM156 | -1 | -1 | 0 | 0 | -1 | -1 | -1 |
| HGF | 1 | 1 | 1 | 1 | 1 | 1 | 0 |
| DKK1 | 1 | 1 | 1 | 1 | 0 | 1 | 0 |
| CTSH | 0 | -1 | -1 | 0 | -1 | -1 | -1 |
| CXCL12 | 1 | 0 | -1 | 0 | -1 | -1 | -1 |
| VCAM1 | 1 | 0 | -1 | 0 | -1 | -1 | -1 |
| IGLC1 | -1 | -1 | 0 | -1 | 0 | -1 | 0 |
| PTPRC | -1 | -1 | -1 | 0 | 0 | -1 | 0 |
| CD19 | -1 | -1 | -1 | 0 | 0 | -1 | 0 |
| LSAMP | 1 | 1 | 1 | 0 | 0 | 1 | 0 |
| HLA-DRB1 | -1 | -1 | -1 | 0 | 0 | -1 | 0 |
| HLA-DPB1 | -1 | -1 | -1 | 0 | 0 | -1 | 0 |
| HLA-DPA1 | -1 | -1 | -1 | 0 | 0 | -1 | 0 |
| LAMP5 | 1 | 1 | 0 | 0 | 0 | 1 | 0 |
| IGLV8-61 | -1 | -1 | -1 | 0 | 0 | 0 | 0 |
| IGLV1-47 | -1 | -1 | -1 | 0 | 0 | 0 | 0 |
| IGLV7-46 | -1 | -1 | -1 | 0 | 0 | 0 | 0 |
| IGLV5-45 | -1 | -1 | -1 | 0 | 0 | 0 | 0 |
| IGLV3-27 | -1 | -1 | -1 | 0 | 0 | 0 | 0 |
| IGHE | -1 | -1 | -1 | 0 | 0 | 0 | 0 |
| IGHV3-15 | -1 | -1 | -1 | 0 | 0 | 0 | 0 |
| IGHV3-72 | -1 | -1 | -1 | 0 | 0 | 0 | 0 |
| IGKV1-27 | -1 | -1 | -1 | 0 | 0 | 0 | 0 |
| PURPL | 1 | 1 | 1 | 0 | 0 | 0 | 0 |
| SLC2A3 | -1 | -1 | 0 | 0 | 0 | -1 | 0 |
| CCND1 | 1 | 1 | 0 | 0 | 0 | 1 | 0 |
| DUSP4 | 1 | 1 | 0 | 0 | 0 | 1 | 0 |
| SWAP70 | -1 | -1 | -1 | 0 | 0 | 0 | 0 |
| MYO1G | -1 | -1 | -1 | 0 | 0 | 0 | 0 |
| PRDM5 | 1 | 1 | 1 | 0 | 0 | 0 | 0 |
| TSPAN7 | 1 | 1 | 0 | 0 | 0 | 1 | 0 |
| PRR15 | 1 | 1 | 1 | 0 | 0 | 0 | 0 |
| NEB | 1 | 1 | 1 | 0 | 0 | 0 | 0 |
| MT1F | -1 | -1 | -1 | 0 | 0 | 0 | 0 |
| IGLV7-43 | -1 | -1 | -1 | 0 | 0 | 0 | 0 |
| IGLV1-40 | -1 | -1 | -1 | 0 | 0 | 0 | 0 |
| IGLV3-19 | -1 | -1 | -1 | 0 | 0 | 0 | 0 |
| IGLV2-18 | -1 | -1 | -1 | 0 | 0 | 0 | 0 |
| IGHV3-23 | -1 | -1 | -1 | 0 | 0 | 0 | 0 |
| IGHV4-34 | -1 | -1 | -1 | 0 | 0 | 0 | 0 |
| IGHV5-51 | -1 | -1 | -1 | 0 | 0 | 0 | 0 |
| IGHV3-66 | -1 | -1 | -1 | 0 | 0 | 0 | 0 |
| IGLV9-49 | -1 | -1 | -1 | 0 | 0 | 0 | 0 |
| IGHV3-64 | -1 | -1 | -1 | 0 | 0 | 0 | 0 |
| LINC01239 | 1 | 1 | 1 | 0 | 0 | 0 | 0 |
| IGKV1-8 | -1 | -1 | -1 | 0 | 0 | 0 | 0 |
| LINC01480 | 1 | 1 | 1 | 0 | 0 | 0 | 0 |
| EDNRB | 1 | 1 | 0 | 1 | 0 | 1 | 0 |
| ADM | 1 | 1 | 0 | 1 | 1 | 0 | 0 |
| BTBD3 | 1 | 1 | 0 | 1 | 0 | 0 | 0 |
| IGKC | 0 | 0 | 0 | -1 | -1 | -1 | -1 |
| IGLV1-44 | -1 | 0 | -1 | -1 | 0 | -1 | 0 |
| MLIP | 1 | 0 | 0 | -1 | 0 | -1 | 0 |
| IGLL5 | -1 | 0 | 0 | -1 | 0 | -1 | 0 |
| CD81 | 0 | -1 | -1 | 0 | -1 | -1 | 0 |
| CD36 | 0 | 0 | -1 | 0 | 0 | -1 | -1 |
| DOCK10 | 0 | 0 | 0 | 0 | -1 | -1 | -1 |
| CTSW | 0 | 0 | 0 | 0 | -1 | -1 | -1 |
| CP | 1 | 0 | -1 | 0 | 0 | 0 | -1 |
| CD5L | 1 | 0 | -1 | 0 | 0 | -1 | 0 |
| FYB1 | 0 | -1 | -1 | 0 | 0 | -1 | 0 |
| LYZ | -1 | 0 | 0 | 0 | 0 | -1 | -1 |
| MS4A6A | 0 | -1 | -1 | 0 | 0 | -1 | 0 |
| TGFBI | 0 | -1 | -1 | 0 | 0 | -1 | 0 |
| TMEM131L | -1 | 0 | -1 | 0 | 0 | 0 | -1 |
| PTPN22 | -1 | 0 | 0 | 0 | 0 | -1 | -1 |
| SLCO2B1 | 1 | 0 | -1 | 0 | 0 | -1 | 0 |
| GPR15 | -1 | 0 | 0 | 0 | -1 | 0 | -1 |
| TXLNB | -1 | 0 | 0 | 0 | -1 | 0 | -1 |
| AXL | 1 | 0 | -1 | 0 | 0 | -1 | 0 |
| RNASE6 | 0 | -1 | 0 | 0 | -1 | -1 | 0 |
| GPR183 | -1 | 0 | -1 | 0 | 0 | -1 | 0 |
| CD14 | 0 | -1 | -1 | 0 | 0 | -1 | 0 |
| MANEA | 0 | -1 | 0 | 0 | -1 | 0 | -1 |
| C1QB | 1 | 0 | -1 | 0 | 0 | -1 | 0 |
| ESRRG | 1 | 0 | 1 | 0 | 0 | 1 | 0 |
| HLA-DRA | 0 | -1 | -1 | 0 | 0 | -1 | 0 |
| IGLV6-57 | -1 | 0 | -1 | 0 | 0 | -1 | 0 |
| CCL3 | 0 | 1 | 0 | 0 | 1 | 0 | 1 |
| IFI6 | 1 | 0 | 0 | 1 | 1 | 0 | 1 |
| NDNF | 1 | 0 | 1 | 1 | 0 | 0 | 1 |
| IFITM1 | 0 | 0 | 0 | 1 | 1 | 0 | 1 |
| ST3GAL6 | 0 | 1 | 1 | 1 | 0 | 0 | 0 |
| H2AC6 | 1 | 0 | 1 | 1 | 0 | 0 | 0 |
| H2BC21 | 1 | 0 | 1 | 1 | 0 | 0 | 0 |
| H1-2 | 0 | 1 | 0 | 1 | 0 | 1 | 0 |
| SELENOP | 0 | 1 | 0 | 1 | 0 | -1 | 0 |

**Supplementary Table 3.** List of the 91 genes that were observed to be differentially expressed between healthy donors and NDMM patients (log2fc ≥ 2 and P-adj < 0,05) in at least 3 of the 7 datasets. 1 represents over-expression in the NDMM group compared to healthy donors. -1 indicates downregulation in the NDMM group. 0 indicates that the gene was not significantly differentially expressed between the 2 groups.

| **GENE NAME** | **GSE153380 P-adj value** | **GSE175384 P-adj value** | **GSE6691 P-adj value** | **GSE116294 P-adj value** | **GSE47552 P-adj value** | **GSE6477 P-adj value** | **GSE16558 P-adj value** | **MEAN P-adj value** |
| --- | --- | --- | --- | --- | --- | --- | --- | --- |
| DKK1 | 1.2E-5 | 1.2E-5 | 0.006 | 0.000004 | X | 0.0001 | X | 0.001 |
| VCAM1 | 0.0004 | X | X | 1.3E-11 | 0.005 | 1.5E-09 | 0.00006 | 0.001 |
| ADM | 0.0005 | 1.1E-5 | 0.004 | X | 0.0004 | X | X | 0.001 |
| CXCL12 | 1.1E-7 | X | X | 0.002 | 0.005 | 0.000002 | 0.001 | 0.002 |
| IFITM1 | X | X | 0.00317 | X | 0.002 | X | 0.0042 | 0.003 |
| PURPL | 8.9E-7 | 0.0001 | X | 0.01 | X | X | X | 0.003 |
| LINC01480 | 0.001 | 0.0004 | X | 0.01 | X | X | X | 0.003 |
| EDNRB | 6.7E-9 | 3.4E-5 | 0.01 | X | X | 0.001 | X | 0.003 |
| CD19 | 0.01 | 0.001 | X | 0.001 | X | 0.00008 | X | 0.003 |
| ST3GAL6 | X | 7.4E-6 | 0.01 | 6.2E-11 | X | X | X | 0.003 |
| TSPAN7 | 5.3E-5 | 0.01 | X | X | X | 0.0001 | X | 0.003 |
| LINC01239 | 2.008E-5 | 0.006 | X | 0.006 | X | X | X | 0.004 |
| IFI6 | 0.003 | X | 0.0004 | X | 0.008 | X | 0.01 | 0.005 |
| CD27 | 4.2E-5 | 1.9E-7 | 0.0002 | 0.03 | 0.009 | 0.000001 | 0.00009 | 0.006 |
| GADD45A | 0.01 | 0.001 | X | 0.02 | 0.000001 | X | 0.0004 | 0.006 |
| HGF | 0.0002 | 1.8E-6 | 0.03 | 0.0008 | 0.007 | 0.001 | X | 0.006 |
| BTBD3 | 0.0005 | 0.02 | 0.002 | X | X | X | X | 0.007 |
| CCL3 | X | 0.009 | X | X | 0.01 | X | 0.007 | 0.008 |
| PRR15 | 0.02 | 0.0008 | X | 0.007 | X | X | X | 0.009 |
| PTPRC | 5.8E-6 | 0.04 | X | 0.01 | X | 0.000003 | X | 0.01 |
| LAMP5 | 0.0004 | 7.4E-6 | X | X | X | 0.04 | X | 0.01 |
| CCND1 | 0.0004 | 0.04 | X | X | X | 0.01 | X | 0.01 |
| MT1F | 0.004 | 0.03 | X | 2.2E-8 | X | X | X | 0.01 |
| CD81 | X | 0.04 | X | 0.01 | 0.0001 | 0.000001 | X | 0.01 |
| TGFBI | X | 0.03 | X | 0.0001 | X | 8.42E-09 | X | 0.01 |
| NDNF | 0.0001 | X | 0.04 | 0.000008 | X | X | 0.01 | 0.01 |
| DUSP4 | 0.03 | 0.02 | X | X | X | 0.000003 | X | 0.01 |
| PRDM5 | 0.02 | 0.01 | X | 0.03 | X | X | X | 0.02 |

**Supplementary Table 4.** P-values (adjusted for multiple testing correction) of the 28 differentially expressed genes across the 7 datasets. X indicates that the gene was not significantly differentially expressed between the 2 groups.

| **GENE NAME** | **Number of patients (%) that over/under-expressed each gene in the GSE153380 dataset** | **Number of patients (%) that over/under-expressed each gene in the GSE175384 dataset** |
| --- | --- | --- |
| CD27 | 14 (100%) | 32 (100%) |
| GADD45A | 7 (50%) | 23 (71%) |
| HGF | 10 (71%) | 28 (87%) |
| DKK1 | 13 (92%) | 26 (81%) |
| CXCL12 | 11 (78%) | 1 (3%) |
| VCAM1 | 10 (71%) | 2 (6%) |
| PTPRC | 11 (78%) | 28 (87%) |
| CD19 | 11 (78%) | 29 (90%) |
| LAMP5 | 7 (50%) | 24 (75%) |
| PURPL | 13 (92%) | 27 (84%) |
| CCND1 | 9 (64%) | 9 (28%) |
| DUSP4 | 8 (57%) | 16 (50%) |
| PRDM5 | 9 (64%) | 26 (81%) |
| TSPAN7 | 10 (71%) | 20 (62%) |
| PRR15 | 9 (64%) | 25 (78%) |
| MT1F | 14 (100%) | 31 (96%) |
| LINC01239 | 13 (92%) | 25 (78%) |
| LINC01480 | 12 (85%) | 26 (81%) |
| EDNRB | 11 (78%) | 27 (84%) |
| ADM | 8 (57%) | 29 (90%) |
| BTBD3 | 10 (71%) | 29 (90%) |
| CD81 | 10 (71%) | 30 (93%) |
| TGFBI | 0 (0%) | 0 (0%) |
| CCL3 | 5 (35%) | 19 (59%) |
| IFI6 | 10 (71%) | 14 (43%) |
| NDNF | 11 (78%) | 19 (59%) |
| IFITM1 | 4 (28%) | 14 (43%) |
| ST3GAL6 | 9 (64%) | 30 (93%) |

**Supplementary Table 5.** Number (%) of NDMM patients of the 2 RNA-seq datasets that significantly over/under-expressed each of the 28 genes (one sample T-Test or Wilcoxon signed-rank Test, P <0.05). Due to inadequate sampling of GSE153380 dataset (healthy donors n=5) we could not employ the Wilcoxon signed-rank test for the genes whose expression measures were not normally distributed. Therefore, we log-transformed the values and then applied a parametric analysis.

| **DRUG** | **MECHANISM OF ACTION** | **TARGETED MM GENE** | **INVESTIGETIONAL-APPROVED** | **SOURCE** |
| --- | --- | --- | --- | --- |
| Framycetin | CXCR4 ANTAGONIST | CXCL12 | APPROVED | DRUGBANK |
| Plerixafor | CXCR4 ANTAGONIST | CXCL12 | APPROVED | DRUGBANK, ChEMBL, IUPHAR/BPS |
| Ibalizumab | CXCR4 ANTAGONIST | CXCL12 | APPROVED | DRUGBANK |
| Baclofen | CXCR4 ALLOSTERIC MODULATOR | CXCL12 | APPROVED | DRUGBANK |
| Motixafortide | CXCR4 INHIBITOR | CXCL12 | APPROVED | DRUGBANK, ChEMBL, IUPHAR/BPS |
| Mavorixafor | CXCR4 ANTAGONIST | CXCL12 | INVESTIGATIONAL | DRUGBANK, ChEMBL, IUPHAR/BPS |
| MSX-122 | CXCR4 ANTAGONIST | CXCL12 | INVESTIGATIONAL | DRUGBANK, ChEMBL |
| Ulocuplumab | CXCR4 INHIBITOR | CXCL12 | INVESTIGATIONAL | DRUGBANK |
| OLAPTESED PEGOL | CXCL12 INHIBITOR | CXCL12 | INVESTIGATIONAL | ChEMBL |
| BALIXAFORTIDE | CXCR4 ANTAGONIST | CXCL12 | INVESTIGATIONAL | ChEMBL, IUPHAR/BPS |
| BURIXAFOR | CXCR4 ANTAGONIST | CXCL12 | INVESTIGATIONAL | ChEMBL |
| CTCE-9908 | CXCR4 ANTAGONIST | CXCL12 | INVESTIGATIONAL | ChEMBL |
| ULOCUPLUMAB | CXCR4 INHIBITOR | CXCL12 | INVESTIGATIONAL | ChEMBL, IUPHAR/BPS |
| [SDF1 P2G](https://www.guidetopharmacology.org/GRAC/LigandDisplayForward?ligandId=8536) | CXCR4 ANTAGONIST | CXCL12 | INVESTIGATIONAL | IUPHAR/BPS |
| [SDF-1, 1-9[P2G]](https://www.guidetopharmacology.org/GRAC/LigandDisplayForward?ligandId=851) | CXCR4 ANTAGONIST | CXCL12 | INVESTIGATIONAL | IUPHAR/BPS |
| [isothiourea-1t](https://www.guidetopharmacology.org/GRAC/LigandDisplayForward?ligandId=2899) | CXCR4 ANTAGONIST | CXCL12 | INVESTIGATIONAL | IUPHAR/BPS |
| [isothiourea-1a](https://www.guidetopharmacology.org/GRAC/LigandDisplayForward?ligandId=2900) | CXCR4 ANTAGONIST | CXCL12 | INVESTIGATIONAL | IUPHAR/BPS |
| [T134](https://www.guidetopharmacology.org/GRAC/LigandDisplayForward?ligandId=852) | CXCR4 ANTAGONIST | CXCL12 | INVESTIGATIONAL | IUPHAR/BPS |
| [vMIP-II](https://www.guidetopharmacology.org/GRAC/LigandDisplayForward?ligandId=768) | CXCR4 ANTAGONIST | CXCL12 | INVESTIGATIONAL | IUPHAR/BPS |
| [TIQ-15](https://www.guidetopharmacology.org/GRAC/LigandDisplayForward?ligandId=9882) | CXCR4 ANTAGONIST | CXCL12 | INVESTIGATIONAL | IUPHAR/BPS |
| [compound 46c](https://www.guidetopharmacology.org/GRAC/LigandDisplayForward?ligandId=9883) | CXCR4 ANTAGONIST | CXCL12 | INVESTIGATIONAL | IUPHAR/BPS |
| [T22](https://www.guidetopharmacology.org/GRAC/LigandDisplayForward?ligandId=854) | CXCR4 ANTAGONIST | CXCL12 | INVESTIGATIONAL | IUPHAR/BPS |
| [CX549](https://www.guidetopharmacology.org/GRAC/LigandDisplayForward?ligandId=9701) | CXCR4 ANTAGONIST | CXCL12 | INVESTIGATIONAL | IUPHAR/BPS |
| Maraviroc | CCR5 ANTAGONIST | CCL3 | APPROVED | DRUGBANK, ChEMBL, IUPHAR/BPS |
| Ibalizumab | CCR5 ANTAGONIST | CCL3 | APPROVED | DRUGBANK |
| AZD4818 | CCR1 INHIBITOR | CCL3 | INVESTIGATIONAL | CheMBL |
| BMS-817399 | CCR1 INHIBITOR | CCL3 | INVESTIGATIONAL | CheMBL, IUPHAR/BPS |
| CCX354 | CCR1 INHIBITOR | CCL3 | INVESTIGATIONAL | CheMBL, IUPHAR/BPS |
| [CP-481,715](https://www.guidetopharmacology.org/GRAC/LigandDisplayForward?ligandId=3497) | CCR1 ANTAGONIST | CCL3 | INVESTIGATIONAL | IUPHAR/BPS |
| [BX 471](https://www.guidetopharmacology.org/GRAC/LigandDisplayForward?ligandId=767) | CCR1 ANTAGONIST | CCL3 | INVESTIGATIONAL | IUPHAR/BPS |
| [CCL4](https://www.guidetopharmacology.org/GRAC/LigandDisplayForward?ligandId=757) | CCR1 ANTAGONIST | CCL3 | INVESTIGATIONAL | IUPHAR/BPS |
| [MLN-3897](https://www.guidetopharmacology.org/GRAC/LigandDisplayForward?ligandId=10750) | CCR1 ANTAGONIST | CCL3 | INVESTIGATIONAL | IUPHAR/BPS |
| [compound 2b-1](https://www.guidetopharmacology.org/GRAC/LigandDisplayForward?ligandId=3696) | CCR1 ANTAGONIST | CCL3 | INVESTIGATIONAL | IUPHAR/BPS |
| [vMIP-II](https://www.guidetopharmacology.org/GRAC/LigandDisplayForward?ligandId=768) | CCR1 ANTAGONIST | CCL3 | INVESTIGATIONAL | IUPHAR/BPS |
| [UCB35625](https://www.guidetopharmacology.org/GRAC/LigandDisplayForward?ligandId=3536) | CCR1 ANTAGONIST | CCL3 | INVESTIGATIONAL | IUPHAR/BPS |
| [CCL18](https://www.guidetopharmacology.org/GRAC/LigandDisplayForward?ligandId=4382) | CCR1 ANTAGONIST | CCL3 | INVESTIGATIONAL | IUPHAR/BPS |
| INCB-9471 | CCR5 ANTAGONIST | CCL3 | INVESTIGATIONAL | DRUGBANK |
| Mavorixafor | CCR5 ANTAGONIST | CCL3 | INVESTIGATIONAL | DRUGBANK |
| Leronlimab | CCR5 ANTAGONIST | CCL3 | INVESTIGATIONAL | DRUGBANK, ChEMBL |
| Aplaviroc | CCR5 ANTAGONIST | CCL3 | INVESTIGATIONAL | DRUGBANK, ChEMBL, IUPHAR/BPS |
| Vicriviroc | CCR5 ANTAGONIST | CCL3 | INVESTIGATIONAL | DRUGBANK, ChEMBL, IUPHAR/BPS |
| CCR5 mAb | CCR5 ANTAGONIST | CCL3 | INVESTIGATIONAL | DRUGBANK |
| CENICRIVIROC | CCR5 ANTAGONIST | CCL3 | INVESTIGATIONAL | CheMBL, IUPHAR/BPS |
| AZD5672 | CCR5 ANTAGONIST | CCL3 | INVESTIGATIONAL | ChEMBL |
| PF-04634817 | CCR5 ANTAGONIST | CCL3 | INVESTIGATIONAL | ChEMBL |
| PF-232798 | CCR5 ANTAGONIST | CCL3 | INVESTIGATIONAL | ChEMBL |
| BMS-813160 | CCR5 ANTAGONIST | CCL3 | INVESTIGATIONAL | ChEMBL |
| INCB-9471 | CCR5 ANTAGONIST | CCL3 | INVESTIGATIONAL | ChEMBL |
| HGS-1025 | CCR5 ANTAGONIST | CCL3 | INVESTIGATIONAL | ChEMBL |
| CCR5MAB004 | CCR5 ANTAGONIST | CCL3 | INVESTIGATIONAL | ChEMBL |
| [ancriviroc](https://www.guidetopharmacology.org/GRAC/LigandDisplayForward?ligandId=804) | CCR5 ANTAGONIST | CCL3 | INVESTIGATIONAL | IUPHAR/BPS |
| [CCL7](https://www.guidetopharmacology.org/GRAC/LigandDisplayForward?ligandId=759) | CCR5 ANTAGONIST | CCL3 | INVESTIGATIONAL | IUPHAR/BPS |
| [TAK-779](https://www.guidetopharmacology.org/GRAC/LigandDisplayForward?ligandId=783) | CCR5 ANTAGONIST | CCL3 | INVESTIGATIONAL | IUPHAR/BPS |
| [AZD5672](https://www.guidetopharmacology.org/GRAC/LigandDisplayForward?ligandId=7686) | CCR5 ANTAGONIST | CCL3 | INVESTIGATIONAL | IUPHAR/BPS |
| [E913](https://www.guidetopharmacology.org/GRAC/LigandDisplayForward?ligandId=3500) | CCR5 ANTAGONIST | CCL3 | INVESTIGATIONAL | IUPHAR/BPS |
| [BMS-681](https://www.guidetopharmacology.org/GRAC/LigandDisplayForward?ligandId=9430) | CCR5 ANTAGONIST | CCL3 | INVESTIGATIONAL | IUPHAR/BPS |
| [CCR5 antagonist 34](https://www.guidetopharmacology.org/GRAC/LigandDisplayForward?ligandId=10106) | CCR5 ANTAGONIST | CCL3 | INVESTIGATIONAL | IUPHAR/BPS |
| [TAK-220](https://www.guidetopharmacology.org/GRAC/LigandDisplayForward?ligandId=800) | CCR5 ANTAGONIST | CCL3 | INVESTIGATIONAL | IUPHAR/BPS |
| [vMIP-II](https://www.guidetopharmacology.org/GRAC/LigandDisplayForward?ligandId=768) | CCR5 ANTAGONIST | CCL3 | INVESTIGATIONAL | IUPHAR/BPS |
| [BMS-753426](https://www.guidetopharmacology.org/GRAC/LigandDisplayForward?ligandId=11597) | CCR5 ANTAGONIST | CCL3 | INVESTIGATIONAL | IUPHAR/BPS |
| [BMS-741672](https://www.guidetopharmacology.org/GRAC/LigandDisplayForward?ligandId=11598) | CCR5 ANTAGONIST | CCL3 | INVESTIGATIONAL | IUPHAR/BPS |
| [MLN-3897](https://www.guidetopharmacology.org/GRAC/LigandDisplayForward?ligandId=10750) | CCR5 ANTAGONIST | CCL3 | INVESTIGATIONAL | IUPHAR/BPS |
| [GSK2239633A](https://www.guidetopharmacology.org/GRAC/LigandDisplayForward?ligandId=10416) | CCR5 ANTAGONIST | CCL3 | INVESTIGATIONAL | IUPHAR/BPS |
| ROX-888 | BINDS TO CCR1/CCR4/CCR5 | CCL3 | INVESTIGATIONAL | DRUGBANK |
| PALBOCICLIB | CYCLIN D1 INHIBITOR | CCND1 | APPROVED | ChEMBL |
| Encorafenib | CYCLIN D1 INHIBITOR | CCND1 | APPROVED | DRUGBANK |
| [Acetylsalicylic acid](https://go.drugbank.com/drugs/DB00945) | CYCLIN D1 DOWNREGULATOR | CCND1 | APPROVED | DRUGBANK |
| BRICICLIB | CYCLIN D1 INHIBITOR | CCND1 | INVESTIGATIONAL | ChEMBL |
| Bryostatin 1 | CYCLIN D1 INHIBITOR | CCND1 | INVESTIGATIONAL | DRUGBANK |
| Arsenic trioxide | CYCLIN D1 INHIBITOR | CCND1 | INVESTIGATIONAL | DRUGBANK |
| Valproic acid | HGF INHIBITOR | HGF | APPROVED | DRUGBANK |
| Cabozantinib | HGFR INHIBITOR | HGF | APPROVED | DRUGBANK, ChEMBL, IUPHAR/BPS |
| Crizotinib | HGFR INHIBITOR | HGF | APPROVED | DRUGBANK, ChEMBL, IUPHAR/BPS |
| Brigatinib | HGFR INHIBITOR | HGF | APPROVED | DRUGBANK |
| Fostamatinib | HGFR INHIBITOR | HGF | APPROVED | DRUGBANK |
| Capmatinib | HGFR INHIBITOR | HGF | APPROVED | DRUGBANK, ChEMBL, IUPHAR/BPS |
| Tepotinib | HGFR INHIBITOR | HGF | APPROVED | DRUGBANK, ChEMBL, IUPHAR/BPS |
| Tivozanib | HGFR INHIBITOR | HGF | APPROVED | DRUGBANK |
| Amivantamab | HGFR INHIBITOR | HGF | APPROVED | DRUGBANK, ChEMBL |
| Sunitinib | HGFR INHIBITOR | HGF | APPROVED | DRUGBANK |
| RILOTUMUMAB | HGF INHIBITOR | HGF | INVESTIGATIONAL | ChEMBL |
| FICLATUZUMAB | HGF INHIBITOR | HGF | INVESTIGATIONAL | ChEMBL |
| Foretinib | HGFR INHIBITOR | HGF | INVESTIGATIONAL | DRUGBANK, ChEMBL, IUPHAR/BPS |
| Amuvatinib | HGFR INHIBITOR | HGF | INVESTIGATIONAL | DRUGBANK, ChEMBL |
| [Tivantinib](https://go.drugbank.com/drugs/DB12200) | HGFR INHIBITOR | HGF | INVESTIGATIONAL | DRUGBANK, ChEMBL, IUPHAR/BPS |
| AMG-208 | HGFR INHIBITOR | HGF | INVESTIGATIONAL | DRUGBANK, ChEMBL |
| TELISOTUZUMAB VEDOTIN | HGFR BINDER | HGF | INVESTIGATIONAL | ChEMBL |
| SAVOLITINIB | HGFR INHIBITOR | HGF | INVESTIGATIONAL | ChEMBL, IUPHAR/BPS |
| ONARTUZUMAB | HGFR INHIBITOR | HGF | INVESTIGATIONAL | ChEMBL |
| AMG-337 | HGFR INHIBITOR | HGF | INVESTIGATIONAL | ChEMBL, IUPHAR/BPS |
| GOLVATINIB | HGFR INHIBITOR | HGF | INVESTIGATIONAL | ChEMBL, IUPHAR/BPS |
| EMIBETUZUMAB | HGFR INHIBITOR | HGF | INVESTIGATIONAL | ChEMBL |
| MK-2461 | HGFR INHIBITOR | HGF | INVESTIGATIONAL | ChEMBL, IUPHAR/BPS |
| MERESTINIB | HGFR INHIBITOR | HGF | INVESTIGATIONAL | ChEMBL, IUPHAR/BPS |
| EMB-01 | HGFR INHIBITOR | HGF | INVESTIGATIONAL | ChEMBL |
| BMS-777607 | HGFR INHIBITOR | HGF | INVESTIGATIONAL | ChEMBL, IUPHAR/BPS |
| SAR-125844 | HGFR INHIBITOR | HGF | INVESTIGATIONAL | ChEMBL |
| GLESATINIB | HGFR INHIBITOR | HGF | INVESTIGATIONAL | ChEMBL, IUPHAR/BPS |
| BMS-817378 | HGFR INHIBITOR | HGF | INVESTIGATIONAL | ChEMBL |
| BPI-9016 | HGFR INHIBITOR | HGF | INVESTIGATIONAL | ChEMBL |
| JNJ-38877605 | HGFR INHIBITOR | HGF | INVESTIGATIONAL | ChEMBL |
| BMS-794833 | HGFR INHIBITOR | HGF | INVESTIGATIONAL | ChEMBL |
| MK-8033 | HGFR INHIBITOR | HGF | INVESTIGATIONAL | ChEMBL |
| BMS-698769 | HGFR INHIBITOR | HGF | INVESTIGATIONAL | ChEMBL |
| SGX-523 | HGFR INHIBITOR | HGF | INVESTIGATIONAL | ChEMBL, IUPHAR/BPS |
| TAS-115 | HGFR INHIBITOR | HGF | INVESTIGATIONAL | ChEMBL |
| ALTIRATINIB | HGFR INHIBITOR | HGF | INVESTIGATIONAL | ChEMBL, IUPHAR/BPS |
| PF-04217903 | HGFR INHIBITOR | HGF | INVESTIGATIONAL | ChEMBL |
| TELISOTUZUMAB | HGFR INHIBITOR | HGF | INVESTIGATIONAL | ChEMBL |
| EMD-1204831 | HGFR INHIBITOR | HGF | INVESTIGATIONAL | ChEMBL |
| ARRY-300 | HGFR INHIBITOR | HGF | INVESTIGATIONAL | ChEMBL |
| NINGETINIB | HGFR INHIBITOR | HGF | INVESTIGATIONAL | ChEMBL, IUPHAR/BPS |
| [SGX-523](https://www.guidetopharmacology.org/GRAC/LigandDisplayForward?ligandId=5709) | HGFR INHIBITOR | HGF | INVESTIGATIONAL | IUPHAR/BPS |
| [PHA-665752](https://www.guidetopharmacology.org/GRAC/LigandDisplayForward?ligandId=5700) | HGFR INHIBITOR | HGF | INVESTIGATIONAL | IUPHAR/BPS |
| [pamufetinib](https://www.guidetopharmacology.org/GRAC/LigandDisplayForward?ligandId=10460) | HGFR INHIBITOR | HGF | INVESTIGATIONAL | IUPHAR/BPS |
| [gumarontinib](https://www.guidetopharmacology.org/GRAC/LigandDisplayForward?ligandId=11623) | HGFR INHIBITOR | HGF | INVESTIGATIONAL | IUPHAR/BPS |
| [ensartinib](https://www.guidetopharmacology.org/GRAC/LigandDisplayForward?ligandId=8959) | HGFR INHIBITOR | HGF | INVESTIGATIONAL | IUPHAR/BPS |
| [elzovantinib](https://www.guidetopharmacology.org/GRAC/LigandDisplayForward?ligandId=11869) | HGFR INHIBITOR | HGF | INVESTIGATIONAL | IUPHAR/BPS |
| [dalmelitinib](https://www.guidetopharmacology.org/GRAC/LigandDisplayForward?ligandId=12088) | HGFR INHIBITOR | HGF | INVESTIGATIONAL | IUPHAR/BPS |
| [D6808](https://www.guidetopharmacology.org/GRAC/LigandDisplayForward?ligandId=12248) | HGFR INHIBITOR | HGF | INVESTIGATIONAL | IUPHAR/BPS |
| [vabametkib](https://www.guidetopharmacology.org/GRAC/LigandDisplayForward?ligandId=12391) | HGFR INHIBITOR | HGF | INVESTIGATIONAL | IUPHAR/BPS |
| [OMO-1](https://www.guidetopharmacology.org/GRAC/LigandDisplayForward?ligandId=10507) | HGFR INHIBITOR | HGF | INVESTIGATIONAL | IUPHAR/BPS |
| [compound 1o](https://www.guidetopharmacology.org/GRAC/LigandDisplayForward?ligandId=8143) | HGFR INHIBITOR | HGF | INVESTIGATIONAL | IUPHAR/BPS |
| [SU11274](https://www.guidetopharmacology.org/GRAC/LigandDisplayForward?ligandId=5057) | HGFR INHIBITOR | HGF | INVESTIGATIONAL | IUPHAR/BPS |
| [compound 27](https://www.guidetopharmacology.org/GRAC/LigandDisplayForward?ligandId=8210) | HGFR INHIBITOR | HGF | INVESTIGATIONAL | IUPHAR/BPS |
| [zanzalintinib](https://www.guidetopharmacology.org/GRAC/LigandDisplayForward?ligandId=12093) | HGFR INHIBITOR | HGF | INVESTIGATIONAL | IUPHAR/BPS |
| [compound 19a](https://www.guidetopharmacology.org/GRAC/LigandDisplayForward?ligandId=10181) | HGFR INHIBITOR | HGF | INVESTIGATIONAL | IUPHAR/BPS |
| [AM7](https://www.guidetopharmacology.org/GRAC/LigandDisplayForward?ligandId=8292) | HGFR INHIBITOR | HGF | INVESTIGATIONAL | IUPHAR/BPS |
| [sitravatinib](https://www.guidetopharmacology.org/GRAC/LigandDisplayForward?ligandId=9920) | HGFR INHIBITOR | HGF | INVESTIGATIONAL | IUPHAR/BPS |
| [compound R-16](https://www.guidetopharmacology.org/GRAC/LigandDisplayForward?ligandId=8213) | HGFR INHIBITOR | HGF | INVESTIGATIONAL | IUPHAR/BPS |
| [compound 16](https://www.guidetopharmacology.org/GRAC/LigandDisplayForward?ligandId=8178) | HGFR INHIBITOR | HGF | INVESTIGATIONAL | IUPHAR/BPS |
| [compound 8i](https://www.guidetopharmacology.org/GRAC/LigandDisplayForward?ligandId=9905) | HGFR INHIBITOR | HGF | INVESTIGATIONAL | IUPHAR/BPS |
| [RIPK3 inhibitor 18](https://www.guidetopharmacology.org/GRAC/LigandDisplayForward?ligandId=10389) | HGFR INHIBITOR | HGF | INVESTIGATIONAL | IUPHAR/BPS |
| [alectinib](https://www.guidetopharmacology.org/GRAC/LigandDisplayForward?ligandId=7739) | HGFR INHIBITOR | HGF | INVESTIGATIONAL | IUPHAR/BPS |
| [vebreltinib](https://www.guidetopharmacology.org/GRAC/LigandDisplayForward?ligandId=11677) | HGFR INHIBITOR | HGF | INVESTIGATIONAL | IUPHAR/BPS |
| BHQ-880 | DKK1 neutralizing moAB | DKK1 | INVESTIGATIONAL | ChEMBL |
| DKN-01 | DKK1 neutralizing moAB | DKK1 | INVESTIGATIONAL | ChEMBL |
| Carvedilol | VCAM1 INHIBITORS | VCAM1 | APPROVED | DRUGBANK |
| Clove oil | VCAM1 INHIBITORS | VCAM1 | APPROVED | DRUGBANK |
| Bosentan | EDNRB/EDNRA INHIBITOR | EDNRB | APPROVED | DRUGBANK, ChEMBL, IUPHAR/BPS |
| Ambrisentan | EDNRB/EDNRA INHIBITOR | EDNRB | APPROVED | DRUGBANK, ChEMBL, IUPHAR/BPS |
| Sitaxentan | EDNRB/EDNRA INHIBITOR | EDNRB | APPROVED | DRUGBANK, ChEMBL |
| Macitentan | EDNRB/EDNRA INHIBITOR | EDNRB | APPROVED | DRUGBANK, ChEMBL, IUPHAR/BPS |
| [clazosentan](https://www.guidetopharmacology.org/GRAC/LigandDisplayForward?ligandId=12286) | EDNRB INHIBITOR | EDNRB | INVESTIGATIONAL | IUPHAR/BPS |
| Tezosentan | EDNRB INHIBITOR | EDNRB | INVESTIGATIONAL | DRUGBANK, ChEMBL |
| Enrasentan | EDNRB INHIBITOR | EDNRB | INVESTIGATIONAL | DRUGBANK, ChEMBL |
| DARUSENTAN | EDNRB INHIBITOR | EDNRB | INVESTIGATIONAL | ChEMBL |
| RG-7636 | EDNRB INHIBITOR | EDNRB | INVESTIGATIONAL | ChEMBL |
| APROCITENTAN | EDNRB/EDNRA INHIBITOR | EDNRB | INVESTIGATIONAL | ChEMBL, IUPHAR/BPS |
| [TAK 044](https://www.guidetopharmacology.org/GRAC/LigandDisplayForward?ligandId=3913) | EDNRB INHIBITOR | EDNRB | INVESTIGATIONAL | IUPHAR/BPS |
| [SB209670](https://www.guidetopharmacology.org/GRAC/LigandDisplayForward?ligandId=3528) | EDNRB INHIBITOR | EDNRB | INVESTIGATIONAL | IUPHAR/BPS |
| [A192621](https://www.guidetopharmacology.org/GRAC/LigandDisplayForward?ligandId=1009) | EDNRB INHIBITOR | EDNRB | INVESTIGATIONAL | IUPHAR/BPS |
| [BQ788](https://www.guidetopharmacology.org/GRAC/LigandDisplayForward?ligandId=1010) | EDNRB INHIBITOR | EDNRB | INVESTIGATIONAL | IUPHAR/BPS |
| [IRL 2500](https://www.guidetopharmacology.org/GRAC/LigandDisplayForward?ligandId=3887) | EDNRB INHIBITOR | EDNRB | INVESTIGATIONAL | IUPHAR/BPS |
| [atrasentan](https://www.guidetopharmacology.org/GRAC/LigandDisplayForward?ligandId=3487) | EDNRB INHIBITOR | EDNRB | INVESTIGATIONAL | IUPHAR/BPS |
| [K-8794](https://www.guidetopharmacology.org/GRAC/LigandDisplayForward?ligandId=9651) | EDNRB INHIBITOR | EDNRB | INVESTIGATIONAL | IUPHAR/BPS |
| [RES7011](https://www.guidetopharmacology.org/GRAC/LigandDisplayForward?ligandId=3911) | EDNRB INHIBITOR | EDNRB | INVESTIGATIONAL | IUPHAR/BPS |
| [Ro 46-8443](https://www.guidetopharmacology.org/GRAC/LigandDisplayForward?ligandId=1011) | EDNRB INHIBITOR | EDNRB | INVESTIGATIONAL | IUPHAR/BPS |
| Atogepant | CALCITONIN GENE-RELATED PEPTIDE TYPE 1 RECEPTOR ANTAGONIST | ADM | APPROVED | DRUGBANK, IUPHAR/BPS, ChEMBL |
| Zavegepant | CALCITONIN GENE-RELATED PEPTIDE TYPE 1 RECEPTOR ANTAGONIST | ADM | APPROVED | DRUGBANK, IUPHAR/BPS |
| Rimegepant | CALCITONIN GENE-RELATED PEPTIDE TYPE 1 RECEPTOR ANTAGONIST | ADM | APPROVED | DRUGBANK, ChEMBL, IUPHAR/BPS |
| Ubrogepant | CALCITONIN GENE-RELATED PEPTIDE TYPE 1 RECEPTOR ANTAGONIST | ADM | APPROVED | DRUGBANK, ChEMBL, IUPHAR/BPS |
| Erenumab | CALCITONIN GENE-RELATED PEPTIDE TYPE 1 RECEPTOR ANTAGONIST | ADM | APPROVED | DRUGBANK, ChEMBL, IUPHAR/BPS |
| Telcagepant | CALCITONIN GENE-RELATED PEPTIDE TYPE 1 RECEPTOR ANTAGONIST | ADM | INVESTIGATIONAL | DRUGBANK, ChEMBL, IUPHAR/BPS |
| Olcegepant | CALCITONIN GENE-RELATED PEPTIDE TYPE 1 RECEPTOR ANTAGONIST | ADM | INVESTIGATIONAL | DRUGBANK, ChEMBL, IUPHAR/BPS |
| BI-44370 | CALCITONIN GENE-RELATED PEPTIDE TYPE 1 RECEPTOR ANTAGONIST | ADM | INVESTIGATIONAL | ChEMBL |
| MK3207 | CALCITONIN GENE-RELATED PEPTIDE TYPE 1 RECEPTOR ANTAGONIST | ADM | INVESTIGATIONAL | ChEMBL |
| [compound 49](https://www.guidetopharmacology.org/GRAC/LigandDisplayForward?ligandId=8992) | CALCITONIN GENE-RELATED PEPTIDE TYPE 1 RECEPTOR ANTAGONIST | ADM | INVESTIGATIONAL | IUPHAR/BPS |
| [HTL22562](https://www.guidetopharmacology.org/GRAC/LigandDisplayForward?ligandId=11113) | CALCITONIN GENE-RELATED PEPTIDE TYPE 1 RECEPTOR ANTAGONIST | ADM | INVESTIGATIONAL | IUPHAR/BPS |
| [α-CGRP-(8-37)](https://www.guidetopharmacology.org/GRAC/LigandDisplayForward?ligandId=700) | CALCITONIN GENE-RELATED PEPTIDE TYPE 1 RECEPTOR ANTAGONIST | ADM | INVESTIGATIONAL | IUPHAR/BPS |
| [compound 8](https://www.guidetopharmacology.org/GRAC/LigandDisplayForward?ligandId=11148) | CALCITONIN GENE-RELATED PEPTIDE TYPE 1 RECEPTOR ANTAGONIST | ADM | INVESTIGATIONAL | IUPHAR/BPS |
| [α-CGRP](https://www.guidetopharmacology.org/GRAC/LigandDisplayForward?ligandId=701) | CALCITONIN GENE-RELATED PEPTIDE TYPE 1 RECEPTOR ANTAGONIST | ADM | INVESTIGATIONAL | IUPHAR/BPS |
| [AM-(22-52)](https://www.guidetopharmacology.org/GRAC/LigandDisplayForward?ligandId=706) | AM1 RECEPTOR ANTAGONIST | ADM | INVESTIGATIONAL | IUPHAR/BPS |
| [AC187](https://www.guidetopharmacology.org/GRAC/LigandDisplayForward?ligandId=689) | AMY_3_ RECEPTOR ANTAGONIST | ADM | INVESTIGATIONAL | IUPHAR/BPS |
| [CT-(8-32)](https://www.guidetopharmacology.org/GRAC/LigandDisplayForward?ligandId=690) | AMY_3_ RECEPTOR ANTAGONIST | ADM | INVESTIGATIONAL | IUPHAR/BPS |

**Supplementary Table 6.** List of the 175 drugs (31 FDA approved and 144 experimental) that target 8 of the 22 upregulated MM gene products (pooled from DRUGBANK, ChEMBL and IUPHAR/PBS databases).
