# Supplementary figures and images for: A comparative analysis of transcriptomics of newly diagnosed multiple myeloma: exploring drug repurposing
Source: Front Oncol. 2024 Apr 16;14:1390105. doi: 10.3389/fonc.2024.1390105 (PMC11058662; doi:10.3389/fonc.2024.1390105)

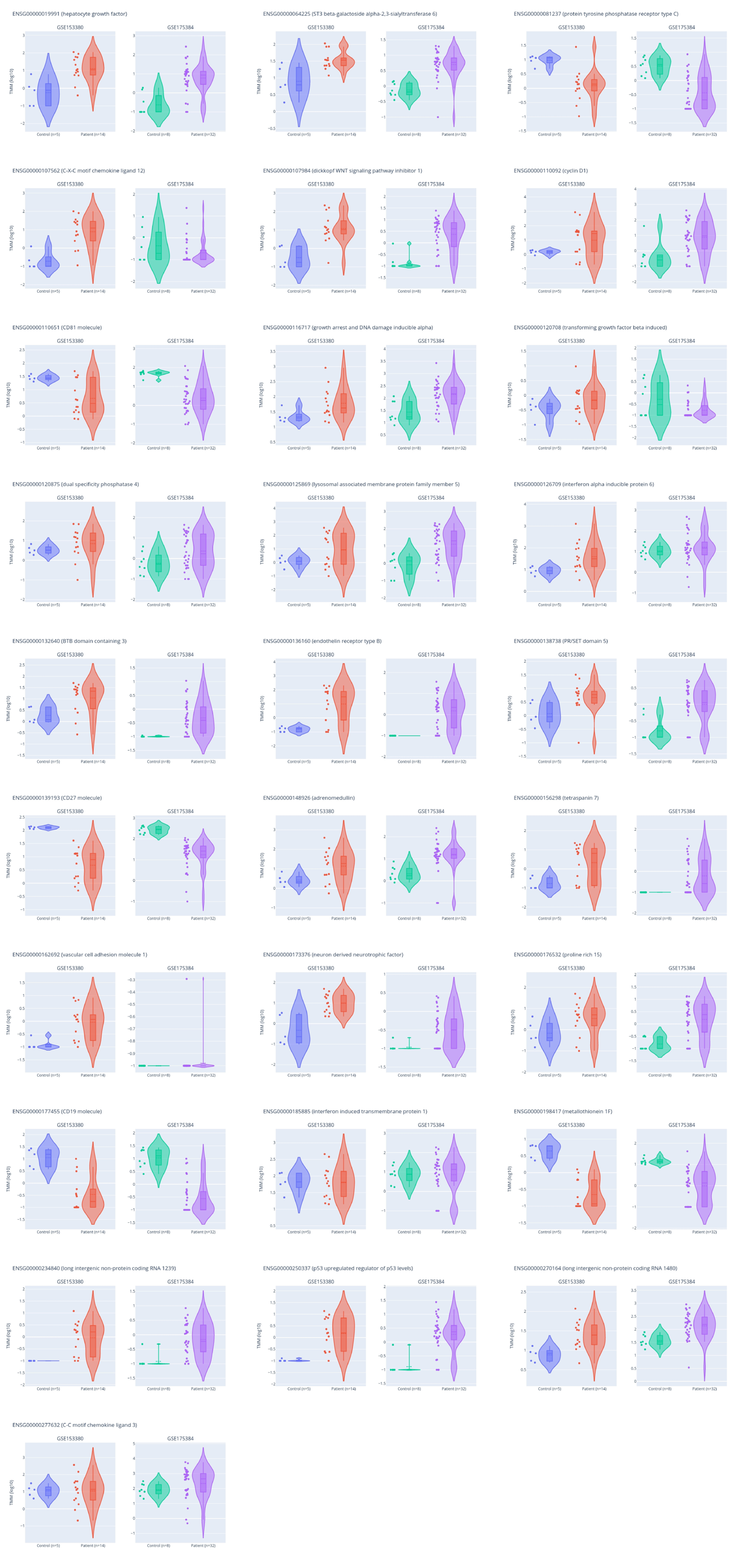

Supplement: Supplementary file 2 [file Image_1.jpeg]
